# Aberrant basal cell clonal dynamics shape early lung carcinogenesis

**DOI:** 10.1126/science.ads9145

**Published:** 2025-06-12

**Authors:** Sandra Gómez-López, Ahmed S.N. Alhendi, Moritz J. Przybilla, Ignacio Bordeu, Zoe E. Whiteman, Timothy Butler, Maral J. Rouhani, Lukas Kalinke, Imran Uddin, Kate E.J. Otter, Deepak P. Chandrasekharan, Marta Lebrusant-Fernandez, Abigail Y.L. Shurr, Pascal F. Durrenberger, David A. Moore, Mary Falzon, James L. Reading, Iñigo Martincorena, Benjamin D. Simons, Peter J. Campbell, Sam M. Janes

**Affiliations:** 1 Lungs for Living Research Centre, UCL Respiratory, University College London; London, WC1E 6JF, UK; 2 Cancer, Ageing and Somatic Mutation Programme, Wellcome Sanger Institute; Hinxton, CB10 1RQ, UK; 3 Departamento de Física, Facultad de Ciencias Físicas y Matemáticas, Universidad de Chile; Santiago, 8370449, Chile; 4 Cancer Research UK City of London Centre Single Cell Genomics Facility, UCL Cancer Institute, University College London; London, WC1E 6DD, UK; 5 Genomics Translational Technology Platform, UCL Cancer Institute, University College London; London, WC1E 6DD, UK; 6 Pre-Cancer Immunology Laboratory, UCL Cancer Institute, University College London; London, WC1E 6DD, UK; 7 Cancer Research UK Lung Cancer Centre of Excellence, University College London Cancer Institute; London, WC1E 6DD, UK; 8 Department of Cellular Pathology, University College London Hospitals NHS Trust; London, NW1 2BU, UK; 9 Department of Applied Mathematics and Theoretical Physics, Centre for Mathematical Sciences, University of Cambridge; Cambridge, CB3 0WA, UK; 10 Gurdon Institute, University of Cambridge; Cambridge, CB2 1QN, UK; 11 Cambridge Stem Cell Institute, Jeffrey Cheah Biomedical Centre, University of Cambridge; Cambridge, CB2 0AW, UK; 12 Department of Haematology, University of Cambridge; Cambridge, CB2 0RE, UK; 13 University College London Hospitals NHS Trust; London, NW1 2BU, UK

## Abstract

Preinvasive squamous lung lesions are precursors of lung squamous cell carcinoma (LUSC). The cellular events underlying lesion formation are unknown. Using a carcinogen-induced model of LUSC with no added genetic hits or cell type bias, we find that carcinogen exposure leads to non-neutral competition among basal cells, aberrant clonal expansions, and basal cell mobilization along the airways. Ultimately, preinvasive lesions develop from a few highly mutated clones that dominate most of the bronchial tree. Multi-site sequencing in human patients confirms the presence of clonally related preinvasive lesions across distinct airway regions. Our work identifies a transition in basal cell clonal dynamics, and an associated shift in basal cell fate, as drivers of field cancerization in the lung.

Lung squamous cell carcinoma (LUSC) evolves from a cancerized field, a population of cells that may show no morphological changes, yet presents some of the phenotypic alterations involved in tumorigenesis, and carries cancer-associated molecular abnormalities (1). The latter are linked to both intrinsic processes, such as ageing, and extrinsic factors, including exposure to mutagens in cigarette smoke (2, 3). With continued damage, increasingly disordered preinvasive lesions become apparent in the bronchial epithelium, half of which ultimately progress to LUSC (4, 5). Elucidating the biological mechanisms underlying the transition of the histologically normal airway into preinvasive disease will inform the design of early cancer interception strategies.

The pseudostratified epithelium lining the adult trachea and mainstem bronchi is composed of basal cells and various populations of luminal cells. During homeostasis, basal cells divide to self-renew and to give rise to luminal cells, either directly or through the generation of intermediate progenitor cells (6–9). Secretory and ciliated cells make up most of the luminal cell compartment, whereas ionocytes, neuroendocrine and tuft (also known as brush) cells are present at low frequencies (6, 7, 10). Secretory cells also have the capacity to self-renew and to produce ciliated cells (11). However, their loss rate is higher than their self-renewal rate, and thus are continuously replenished by basal cells (8).

Defining markers of basal cells, including Keratin 5 (KRT5) and the transcription factor p63, are also expressed in LUSC. Their expression profile and capacity for long-term self-renewal have highlighted basal cells as the suspected cell-of-origin of LUSC (12). Indeed, we and others have shown that tobacco smoking—the main lung cancer risk factor (13, 14)—markedly increases both mutational burden and the presence of cancer-driver mutations in basal cells from the histologically normal human bronchial epithelium (2, 3). Clonal tracking in the normal human airway and during early stepwise carcinogenesis is challenging. Analyses require the ability to sample extremely small regions across a spectrum of lesion grades and/or longitudinally, both impractical in conventional clinical practice.

Murine models offer a tractable platform to study cancer initiation and progression. To date, investigation of the cellular origin of LUSC has relied mainly on the use of genetically-engineered mouse models (GEMMs) with cell type-restricted genetic alterations (15, 16). While these studies have demonstrated the ability of distinct airway progenitor cells to produce tumors when targeted with specific oncogenic hits, it is unclear whether the same cell populations would do so in response to environmental mutagens (17). Additionally, when early disease stages were examined, only a minority of GEMMs displayed a squamous phenotype throughout disease development (12, 18). Chemical carcinogenesis models provide an alternative to GEMMs with the advantage of exhibiting a high mutational burden, more closely resembling the complexity of human cancers (17). N-nitroso-tris-chloroethylurea (NTCU), a DNA alkylating agent, induces the development of both preinvasive squamous lung lesions and invasive LUSC in mice, allowing examination of squamous cell lung carcinogenesis from its earliest stages (19, 20).

Here, we used lineage tracing, whole-mount imaging, as well as single-cell RNA and low-input whole-genome sequencing (scRNA-seq, WGS) to track basal cell trajectories following NTCU exposure. Our results demonstrate a lineage relationship between basal cells and squamous lung lesions in vivo. Biophysical modeling reveals that carcinogen exposure leads to early non-neutral basal cell competition in the tracheobronchial epithelium, which eventually drives clone dominance in the peripheral airways. This change in clonal dynamics is associated with a basal cell fate shift, also identified in the airway of smokers. In mouse and human, we demonstrate evidence of clonal relatedness between preinvasive lesions across distinct anatomical sites, detailing mechanistic insights into aberrant clonal expansions and cell migration contributing to field cancerization.

## NTCU-induced squamous lung lesions originate from pre-existing basal cells

**Figure 1:**
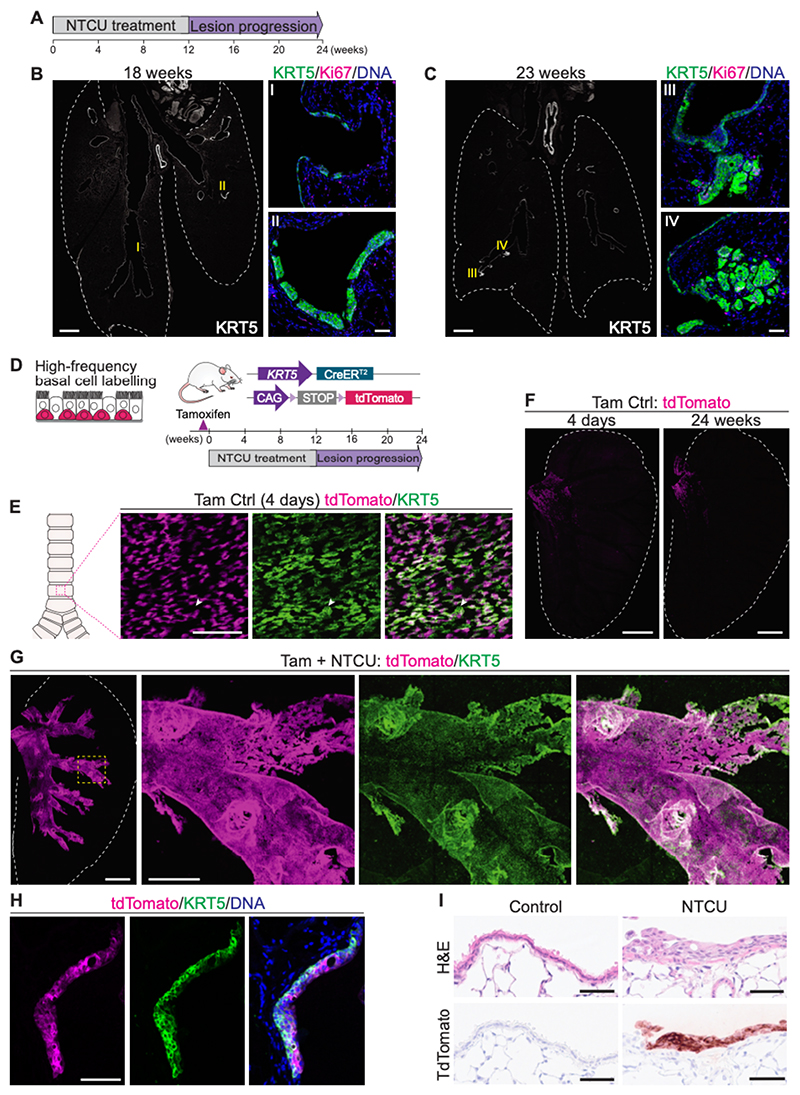
NTCU-induced squamous lung lesions develop from pre-existing basal cells. **(A)** NTCU administration protocol for the induction of murine lung squamous cell carcinoma. **(B)** Immunofluorescence for the basal cell and squamous cell lesion and tumor marker KRT5 and the proliferation marker Ki67 on murine lung sections 18 weeks after NTCU treatment commencement. Tissue overview shows abnormal KRT5 expression in the bronchial tree. Scale bar, 1 mm. (I, II) Low-grade preinvasive lesions are shown. Scale bar, 50 μm. **(C)** Immunostaining for KRT5 and Ki67 on lung sections from NTCU-treated mice 23 weeks after treatment commencement. Tissue overview shows regions in the bronchial tree expressing KRT5. Scale bar, 1 mm. (III, IV) Invasive tumors filling intraparenchymal spaces are shown. Scale bar, 50 μm. **(D)** Strategy to track lineage-labeled airway basal cells during NTCU-induced lung carcinogenesis in *KRT5-CreER;tdTomato* mice. **(E)** 3D projection of dorsal trachea whole-mount showing expression of tdTomato in the great majority of KRT5^+^ basal cells following tamoxifen treatment. Arrow points to non-labeled KRT5^+^ cell. Scale bar, 100 μm. **(F)** Left lung lobe whole-mounts showing lineage-traced tdTomato^+^ cells in control lungs. Scale bar, 2 mm. **(G)** 3D projection of a lung whole-mount from a NTCU-treated mouse. Lineage-labeled tdTomato^+^ cells co-expressing KRT5 are detected throughout the bronchial tree. Scale bars, 2 mm (overview) and 500 μm (magnified region). **(H)** Immunostaining for tdTomato and KRT5 on bronchial section collected 18 weeks after NTCU start. Histology is indicative of squamous dysplasia, with partially disorganized layers of epithelial cells. Scale bar, 100 μm. **(I)** Bronchial tissue sections from control and NTCU-treated *KRT5-CreER;tdTomato* mice stained with hematoxylin and eosin (H&E, top), or processed for tdTomato immunohistochemistry (bottom). Differentiated luminal cells are seen in the control bronchial epithelium, and no lineage-labeled cells are detected. Following NTCU administration, tdTomato^+^ dysplastic lesions and loss of luminal cells are observed. Scale bars, 50 μm.

Topical administration of NTCU to mice for 12 weeks leads first to the formation of preinvasive lung lesions along the bronchial tree, and eventually to the development of invasive lung squamous cell carcinoma within the next 12 weeks (Figure 1A-C) (20). The histology and progressive nature of this murine model closely mimics human LUSC, making it an ideal system to track the cellular origin of the disease.

To assess the contribution of basal cells to NTCU-induced carcinogenesis in vivo we used lineage tracing. Unlike the human respiratory tract, where basal cells are found throughout the conducting airways, including the bronchioles, in the mouse they are mainly confined to the extrapulmonary airways (9). *Tg(KRT5-CreER);R26R-tdTomato* mice (hereafter referred to as *KRT5-CreER;tdTomato*) received tamoxifen for five consecutive days to label airway basal cells at high density (Figure 1D). We confirmed expression of tdTomato in the great majority of KRT5^+^ tracheal basal cells by whole-mount immunostaining four days after the last tamoxifen dose (Figure 1E). Mice were then treated with NTCU for 12 weeks, and the tracheobronchial tree examined in tissue whole-mounts 18-24 weeks post-tamoxifen (Figure 1F-G; Figure S1A). In control mice treated only with tamoxifen, tdTomato^+^ cells remained restricted to the proximal end of the main bronchus throughout the 24-week period, consistent with the compartmentalization of the epithelium (Figure 1F). In the NTCU-treated group, however, cells co-expressing tdTomato and KRT5 were evident in the main bronchus and bronchioles (Figure 1G, Figure S1B,C). Histological analyses of sections across the tdTomato^+^KRT5^+^ bronchial epithelium confirmed the presence of preinvasive disease, ranging from flat atypia to squamous dysplasia (Figure 1H,I). All the lesions identified across 8 different individuals examined were tdTomato^+^. These findings demonstrate that NTCU-induced squamous lung lesions derived from lineage-labeled basal cells. Notably, lineage-tracing of *Scgb1a1*-expressing secretory cells revealed no contribution of this population to NTCU-induced disease (Figure S2).

## NTCU induces non-neutral mutant clone expansions

**Figure 2:**
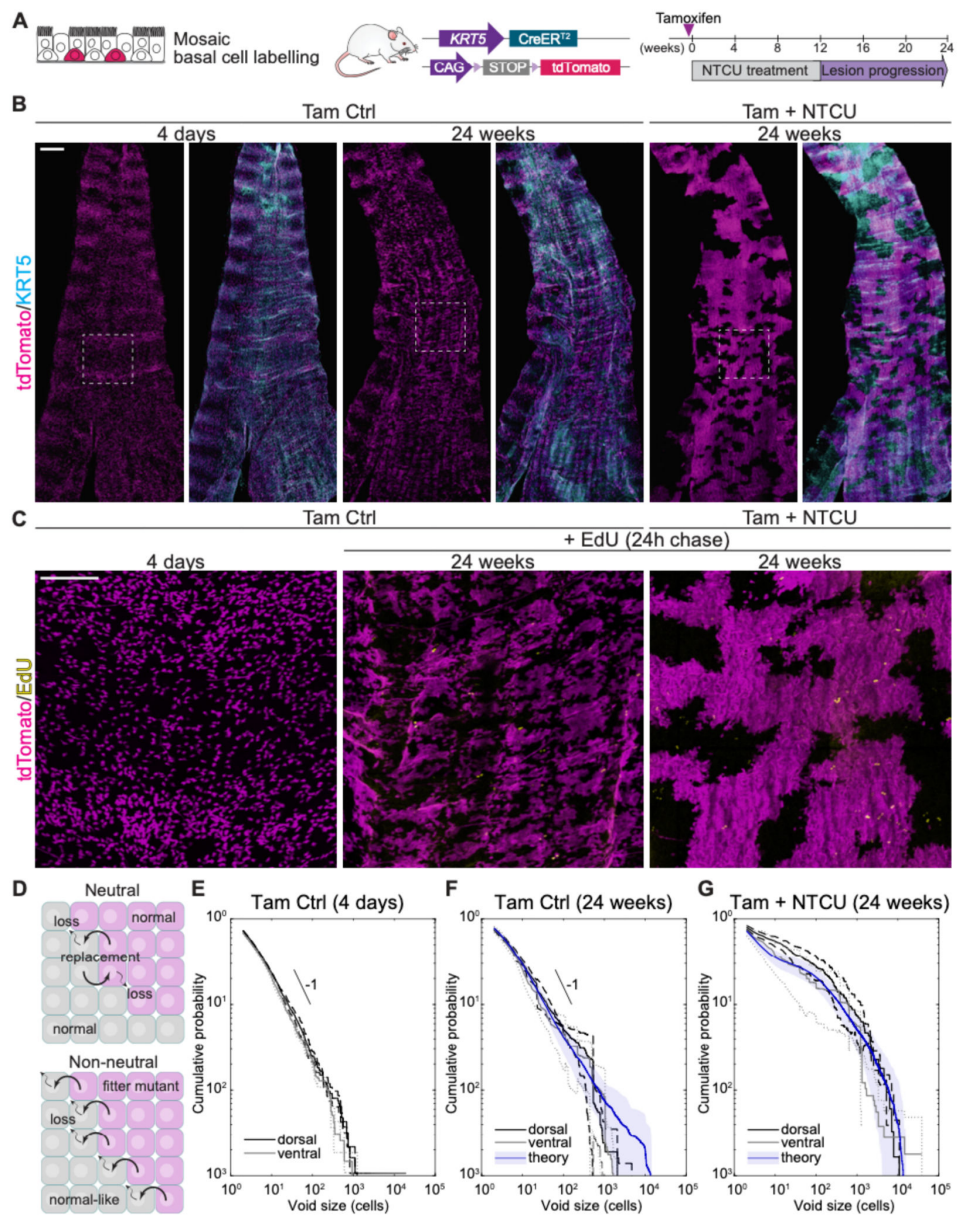
Carcinogen exposure induces non-neutral basal cell clonal expansions. **(A)** Strategy to track basal-cell derived clones during NTCU-induced lung carcinogenesis using mosaic cell labeling in *KRT5-CreER;tdTomato* mice. **(B)** 3D projections of dorsal trachea whole-mounts from control and NTCU-treated mice at 4 days and 24 weeks post-tamoxifen. The boxed regions highlight the dorsal smooth muscle, running longitudinally between the open cartilage rings, whose dorsal ends can be seen at the lateral edges of the preparation. Scale bar, 500 μm. **(C)** Images of the dorsal tracheal epithelium in the regions indicated in dashed boxes in (B). EdU staining was performed on 24-week samples. Scale bar, 200 μm. **(D)** Schematics of the biophysical models describing the dynamic of the basal cell layer in the control (top) and NTCU-treated trachea (bottom). **(E)** Cumulative probability of observing voids larger than a given size in the control trachea 4 days post-tamoxifen. The black reference line corresponds to a power-law decay with exponent -1. **(F)** Cumulative probability of observing voids larger than a given size in the control trachea 24 weeks post-tamoxifen. Data from 4 individuals in shown (black and grey lines). The blue ‘theory’ line corresponds to the average with shaded standard deviation obtained from 200 numerical simulations of a neutral competition model. The black reference line corresponds to a power-law decay with exponent -1. **(G)** Cumulative probability of observing voids larger than a given size in the trachea of NTCU-treated mice, 24 weeks post-tamoxifen. Data from 4 NTCU-treated individuals is shown (black and grey lines). The blue ‘theory’ line corresponds to the average with shaded standard deviation obtained from 200 numerical simulations of non-neutral model (see Supplementary Text).

To elucidate how carcinogen exposure alters airway basal cell behavior, we used mosaic cell labeling. *KRT5-CreER;tdTomato* mice were given one dose of tamoxifen 4 days before undergoing 12-week NTCU treatment (Figure 2A). Control animals received tamoxifen only. The dorsal and ventral halves of the trachea were examined as whole-mounts 24 weeks after tamoxifen administration (Figure 2B, Figure S3A). In controls, tdTomato-labeled basal cells mostly produced discrete clones along the proximo-distal axis of the trachea. Scattered single cells were also evident (Figure 2C). In contrast, NTCU treatment led to formation of large tdTomato^+^ patches that merged with each other. In both groups, clone boundaries in the dorsal trachea frequently followed the longitudinal smooth muscle bands. Clones over the ventral cartilage, however, had more diffuse margins, creating a less fragmented pattern after NTCU treatment (Figure S3A).

We next applied biophysical modeling to investigate basal cell clonal dynamics in both the control and NTCU-treated groups, focusing on the tracheal epithelium up to but excluding the carina. During homeostasis, basal cells undergo a process of stochastic self-renewal with cell duplication perfectly balanced by the differentiation and loss of neighbors from the basal cell layer (8). To model this dynamic, we turned to a minimal modeling scheme based on a neutral ‘voter’ model type process. In this model, the basal cell layer is represented as a lattice of basal cells in which stochastic loss through differentiation is compensated by the duplication of a neighboring basal cell (Figure 2D). To mimic the mosaic labeling of the *KRT5-CreER;tdTomato* trachea, cells in the lattice model were seeded with spatial distribution matching that of the 4-day labeling control (Figure S3B). We then compared stochastic simulations of the voter model dynamics with the measured distributions of the unlabeled cell clusters, which we refer to as 'voids' (Figure S3C). Focusing on regions larger than one cell, quantitative analysis of the cumulative distribution of void sizes in the dorsal and ventral trachea of the control group showed evidence of a power law-like dependence with an exponent of around -1, as expected for a tissue maintenance process based on neutral competition between basal cells (21). This dynamic showed excellent agreement with the experimental observations at 24 weeks post-labeling (R^2^=0.96 for both dorsal and ventral data) (Figure 2F; Figure S3C; Supplementary Text). Small deviations from this trend could be explained by the inhomogeneous mosaic labeling of the tissue. In contrast, in the NTCU-treated samples there was a shift in the observed distribution, which diverged from the characteristic power law behavior of the neutral model, with an increased fraction of large voids (Figure 2G). As NTCU treatment likely affects the whole basal cell population, neutral competition could in principle be maintained if NTCU caused a global shift in cell behavior. However, numerical simulations revealed that a global increase in cell proliferation, or changes in the fraction or distribution of labeling, were not sufficient to account for the shift in the void size distribution. Indeed, EdU incorporation analyses indicated no statistically significant differences in the number of cycling cells per unit area between groups (Figure 2C; Figure S3E).

We hypothesized that NTCU treatment introduced heterogeneity in the system by altering the cell fate and impairing differentiation of only a subset of basal cells. To test this, we considered a model dynamics in which a small fraction of *fitter mutant* basal cells is able to displace neighboring normal-like mutant cells as they undergo symmetric cell divisions (Figure 2D, bottom panel; Supplementary Text). This results in non-neutral competition; whereby fitter mutant clones colonize tissue at the expense of normal-like cells. In this model, fitter mutant clones compete non-neutrally with normal-like clones, yet they compete neutrally with each other. Considering a small fraction of fitter mutant clones distributed randomly in the system, numerical simulations of the model recapitulated the large patches of labeled and unlabeled regions, providing good agreement between the model and the experimental data (R^2^=0.91 and R^2^=0.90 for dorsal and ventral data, respectively) (Figure 2G; Figure S3D).

Altogether, these results suggest that carcinogen exposure introduces heterogeneity to the basal cell compartment, by shifting the behavior of a fraction of basal cells, leading to their colonization of the tracheal epithelium.

## Carcinogen exposure leads to a basal cell fate shift in the airway epithelium

**Figure 3:**
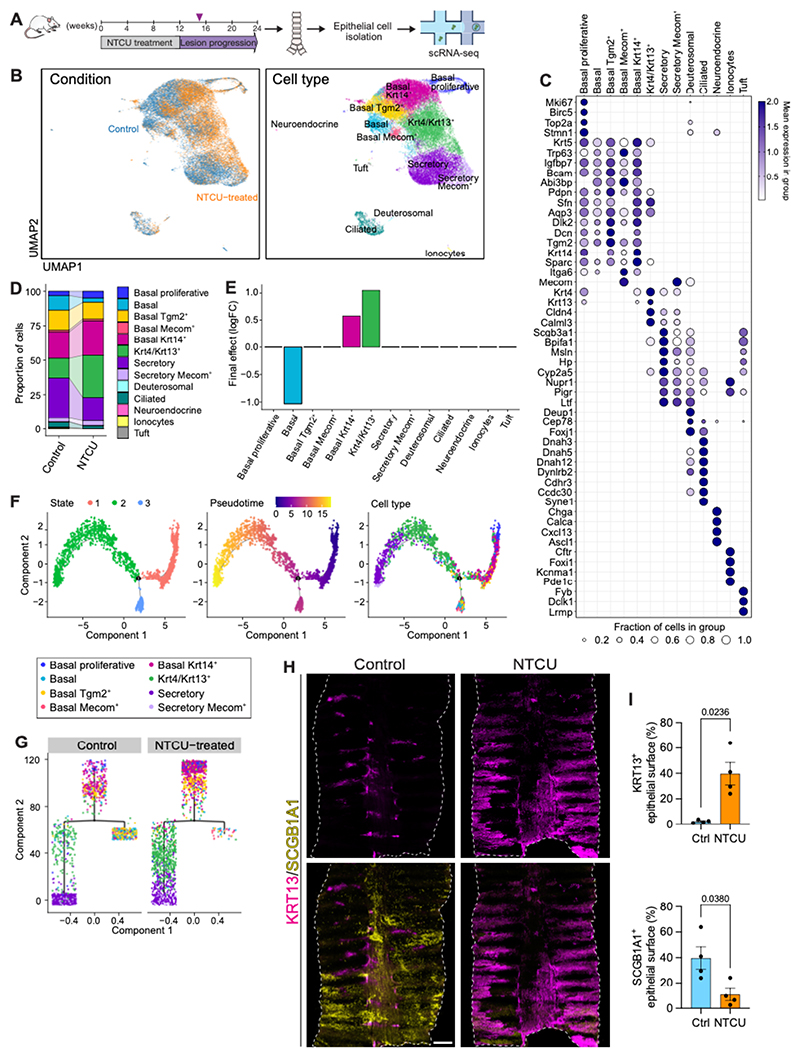
Single cell profiling reveals an epithelial cell fate shift following NTCU treatment. **(A)** Experimental overview of single-cell RNA (scRNA-seq) profiling of the tracheal epithelium of NTCU-treated and age-matched control mice. **(B)** UMAP visualizations colored according to condition (left) and cell type (right) for all mice, depicting 29,088 cells. **(C)** Dotplot depicting the expression of selected marker genes for cell types shown in B. **(D)** Barplot showing changes in relative abundance of tracheal epithelial cell types in the NTCU-treated and control groups, 15 weeks after treatment commencement. **(E)** Barplot showing log 2-fold changes (log_2_FC) in abundance of each cell type between NTCU-treated and control mice calculated using scCODA. Statistical significance was determined by a false discovery rate (FDR) < 0.05 (Benjamini–Hochberg adjusted). **(F)** Trajectory analysis of basal, *Krt4/Krt13*^+^, and secretory cell clusters identified a bifurcated structure, with one major branching point and three cell major cell states. Pseudotime progression is shown in the center. The origin (cell state 1) is enriched in proliferative and basal *Krt14*^+^ cells. The two branches diverge into different cell fates: one dominated by *Krt4/Krt13*^+^ and secretory cells (cell state 2), and the other by basal and basal *Tgm2*^+^ cells (cell state 3). **(G)** Tree structure of the trajectory shown in F, visualizing the distribution and relative abundance of cell types over each branch of the tree structure in the control and NTCU-treated groups. Trajectories were reconstructed in four dimensions but are rendered in two dimensions. **(H)** Immunofluorescence for KRT13 and the secretory cell marker SCGB1A1 on trachea whole-mounts from control and NTCU-treated mice, 18 weeks after NTCU treatment commencement. A single longitudinal cut was done along the ventral tracheal wall to expose the entire epithelial surface. Scale bar, 500 μm. **(I)** Quantitative assessment of the tracheal epithelial surface expressing KRT13 (top) and the secretory marker SCGB1A1 (bottom) in control and NTCU-treated mice, 18 weeks after NTCU treatment commencement. Bars depict mean ± standard error of the mean (SEM). Each dot represents a different individual; *p* values are indicated (unpaired two-tailed *t*-tests with Welch’s correction).

To investigate the consequences of NTCU exposure across the different cell types of the pseudostratified airway epithelium and to gain insights into the mechanisms underlying the change in basal cell dynamics, we conducted scRNA-seq. Tracheal cells were isolated from NTCU-treated mice 3 weeks after treatment completion (15 weeks after treatment commencement). Cells from non-treated age-matched mice were used as controls (Figure 3A). Across 6 mice, a total of 30,020 cells were retained for analysis following quality control (Figure S4A, Data S1). Based on the expression of canonical marker genes, we recapitulated the overarching cellular identities of all clusters, dividing them into epithelial cells (*Epcam, Krt5, Trp63*), macrophages (*Il1b, Mpeg1, Cd68*), and T cells (*Itk, Cd3e, Cd3d*) (Figure S4A,B). There was no evidence of batch effects, with cells clustering according to cellular identity rather than individual (Figure S4A).

We next sub-clustered the epithelial cell fraction (29,088 cells) independently from the immune cell compartment (Figure 3B). Cell type reference signatures were collected from mouse airway single-cell studies and used for identification of individual epithelial cell types (Figure 3B,C; Figure S4C; Data S2, S3). We confirmed the presence of previously described cell types of the upper airways (6, 7, 22), including basal, *Krt4/Krt13*^+^, club/secretory, deuterosomal, ciliated, neuroendocrine, and tuft cells, as well as ionocytes. In line with the previously reported heterogeneity within the murine basal cell compartment (7, 8, 23), we identified five basal cell clusters (Figure 3B,C; Data S3). These included a ‘basal proliferative’ cell subpopulation (*Mki67, Stmn1, Birc5*, and *Top2a*), and a cell fraction characterized by high *Tgm2, Dcn* and *Dlk2* expression, labeled ‘basal Tgm2^+^’. Two subpopulations could be distinguished from the remaining basal cells based on their levels of *Krt14* expression; a ‘basal’ fraction exhibited negligible *Krt14* levels, whereas a ‘basal Krt14^+^’ cluster showed high *Krt14* expression. Lastly, a fifth, smaller basal cell subpopulation displayed high levels of *Trp63* and *Mecom*.

To identify potential NTCU-induced changes in cellular composition within the tracheal epithelium, we performed differential abundance analysis using scCODA (24). NTCU exposure led to a shift within the basal cell pool, with a significant decrease of basal cells lacking expression of *Krt14* and a concomitant increase of *Krt14*^+^ basal cells (FDR < 0.05) (Figure 3D,E, Figure S4D). This change was accompanied by an expansion of the *Krt4/Krt13*^+^ cell fraction following NTCU treatment (FDR < 0.05). Immunostaining analyses of the tracheal epithelium confirmed upregulation of KRT14, accumulation of KRT13^+^ cells across basal and suprabasal locations, and revealed a substantial loss of SCGB1A1^+^ secretory cells 15 weeks after NTCU treatment commencement (Figure S4E,F).

*Krt14* becomes upregulated in the airway epithelium after injury (25), but its persistent expression has been associated with dysregulated repair and preinvasive disease (26), including NTCU-induced tracheal dysplasia, reported to precede the development of bronchial dysplasia in this model (27). To gain insights into the epithelial cell dynamics leading to the observed changes in cell composition, we used Monocle (28) to conduct a trajectory analysis, focusing on the basal, *Krt4/Krt13*^+^, and secretory cell clusters. This analysis orders cells based on their relative gene expression, assuming different cell states along a developmental trajectory. Pseudotime analysis unveiled a biologically plausible progression starting from a state dominated by proliferative and *Krt14*^+^ basal cells (cell state 1), transitioning to either *Krt4/Krt13*^+^ cells and secretory cells (cell state 2) or to basal cells (cell state 3) (Figure 3F). This trajectory is consistent with the known outcomes of basal cell divisions leading to either differentiation or self-renewal, and with the notion that *Krt4/Krt13*^+^ cells constitute a transitional state along one of the possible paths from basal to secretory differentiation (6–8). Visualization of cell type distribution and abundance over each branch of the trajectory revealed that, when compared to controls, in the NTCU-treated group there was, first, an enrichment of *Krt14*^+^ basal cells at the onset of the path, and second, an accumulation of *Krt4/Krt13*^+^ cells along the ‘differentiation’ branch, with fewer secretory cells (Figure 3G; Figure S4G). Whole-mount analyses on tissues obtained 6 weeks after NTCU treatment completion demonstrated a significant increase of KRT13 expression (*p*=0.0236) and decreased SCGB1A1 immunoreactivity (*p*=0.0380) in the tracheal epithelium, in comparison to age-matched controls (Figure 3H-I). Since SCGB1A1^+^ secretory cells act as progenitors of ciliated cells (8, 11), we assessed changes in this population. Expression of the ciliated cell marker FOXJ1 was significantly reduced following NTCU treatment (*p*=0.0348) (Figure S5).

Together, these data indicate that NTCU treatment shifts basal cell fate and negatively affects differentiation, providing support to our biophysical model.

## Epithelial cell fate shift during early human squamous cell lung carcinogenesis

**Figure 4:**
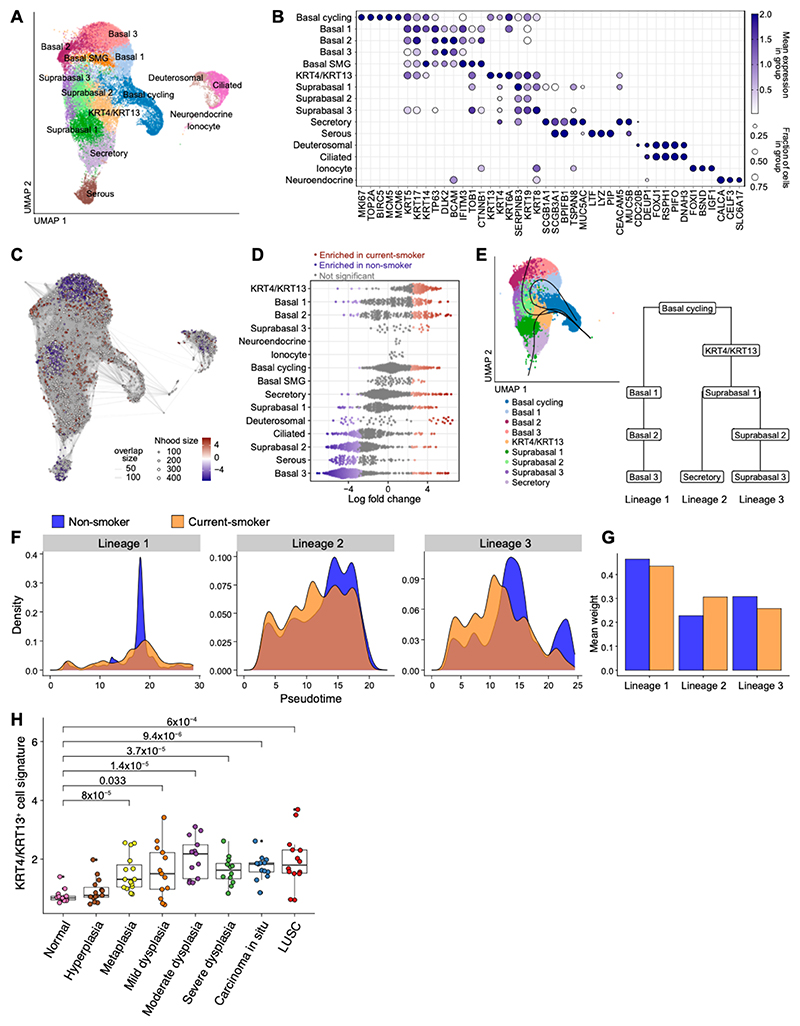
Epithelial cell shift during early human lung squamous cell carcinogenesis. **(A)** UMAP visualization of epithelial cells in the human trachea. Clusters are colored according to cell type. **(B)** Dotplot displaying expression of selected markers for identified human tracheal epithelial cell populations. SMG, submucosal gland. **(C)** Neighborhood graph displaying outcome of differential cell abundance test with MiloR. Neighborhoods (nodes) are colored according to log fold changes between smoking status. **(D)** Beeswarm plot showing differences in tracheal epithelial cell abundance in log fold change between non- and current-smokers. Neighborhoods with differential cell abundance at FDR < 0.1 are colored in blue or red, if enriched in non-smokers or current-smokers, respectively. **(E)** Cell lineage inference for basal, suprabasal, and secretory cell populations from the surface airway epithelium using Slingshot. Principal curves are depicted on the UMAP to the left. The tree on right shows the cell populations in each lineage. Note that *KRT4/KRT13*^+^ cells are identified as a transitional cell state. **(F)** Pseudotime distribution of the three cell lineages identified in the surface airway epithelium, displaying differences between non- and current-smokers. **(G)** Plot depicting mean lineage weights. Weights assignments denote the probability of each cell belonging to a particular lineage. Cell fate choice between lineage 2 and 3 varies between non- and current-smokers. **(H)** *KRT4/KRT13* signature score in normal airway epithelium, increasing grades of lung preinvasive squamous cell lesions and LUSC. Median, upper, and lower quartiles are shown. Individual samples are represented as dots; *p* values are displayed (Wilcoxon test).

We next explored the effects of carcinogen exposure in the histologically normal human airway epithelium. Tracheal brushes were obtained from 3 never- and 3 current-smokers (Data S4) and profiled using scRNA-seq. This dataset was integrated with publicly available data of tracheal biopsies from two additional cohorts, one including 6 never- and 6 current-smokers (29), and another comprising 9 healthy non-smokers (10) (Figure S6A). A total of 45,959 epithelial cells were used for downstream analyses. Cell types were annotated using previously described signatures (10, 29–33) (Data S5), leading to identification of basal, suprabasal, secretory, serous, deuterosomal, ciliated, and neuroendocrine cells, as well as ionocytes (Figure 4A,B; Figure S6B,C; Data S6). Varying degrees of heterogeneity were detected within the basal and suprabasal cell populations (Figure 4A,B). A *KRT4/KRT13*^+^ cell subpopulation was also identified (Figure 4A,B).

For an overall assessment of the effects of cigarette exposure, we conducted a differential cell abundance analysis using MiloR (34). This indicated an enrichment of *KRT4/KRT13*^+^ cells in smokers (Figure 4C,D). To delineate the relationship between the *KRT4/KRT13*^+^ cell state and basal, suprabasal and secretory populations in the human airway surface epithelium we used Slingshot to infer lineage trajectories (35). With the cycling basal cell cluster specified as the origin, three different cell lineages were identified (Figure 4E). Lineage 1 corresponded to self-renewing basal cells. Both lineage 2 and lineage 3 transitioned through the *KRT4/KRT13* state. However, while lineage 2 followed a path towards secretory cell differentiation, lineage 3 progressed through different suprabasal cell states (Figure 4E). Pseudotime distribution analysis throughout lineages indicated slower progression along lineages 2 and 3 in smokers relative to non-smokers, with higher cell densities up to the midpoint of the trajectories and fewer cells reaching the end of the paths (Figure 4F). To evaluate cell fate choice differences by smoking status, we compared mean lineage weights, which represent the relative contribution of each cell to a specific lineage. This revealed a shift from lineage 3 to lineage 2 in smokers (FDR < 5.4x10^-23^; Figure 4G), resulting from an increased proportion of *KRT4/KRT13*^+^ cells and a concomitant reduction of suprabasal cells in the epithelium of smokers. Therefore, both in mouse and human, carcinogen exposure leads to differential cell fate choice and to accumulation of cells in a transitional *KRT4/KRT13*^+^ state in the airway epithelium.

We then derived a *KRT4/KRT13* gene expression signature based on the top 50 differentially expressed genes identified in our scRNA-seq analyses, and evaluated its expression in a bulk RNA-seq dataset including 122 human bronchial biopsies from 77 patients, ranging from histologically normal airway throughout increasing stages of preinvasive disease up to LUSC (36). We found a significant enrichment of the *KRT4/KRT13* signature in preinvasive lesions, from metaplasia to carcinoma in situ (CIS), as well as in invasive tumors, when compared to normal (metaplasia *p*=8x10^-5^; mild dysplasia *p*=0.0033; moderate dysplasia *p*=1.4x10^-5^, severe dysplasia *p*=3.7x10^-5^, CIS *p*=9.4x10^-6^, LUSC *p*=6x10^-4^) (Figure 4H). This supports the relevance of this cell state in preinvasive disease development.

## Mutagenic consequences of N-nitroso-tris-chloroethyl urea in the airway epithelium

NTCU belongs to the class of nitrosourea compounds, DNA alkylating agents which are often used in chemotherapy, similar to platinum-based chemotherapeutic drugs like cis- or carbo-platin or non-classical alkylating agents such as temozolomide. Alkylating agents have been shown to induce cytotoxic and mutagenic adducts onto DNA, leaving mutagen-specific signatures of DNA damage (37). We used laser-capture microdissection (LCM) followed by low-input whole-genome sequencing (WGS) (38) to profile the genomic consequences of NTCU on epithelial cells across the bronchial tree. A total of 142 epithelial microbiopsies along the trachea and intrapulmonary airways were obtained from two mice, MD6812 and MD7047, 23 and 24 weeks after NTCU commencement, respectively. Low-input WGS was performed to a median depth of 24x (range of 8x−46x), enabling us to investigate the burden of somatic substitutions and small insertions and deletions (indels; Data S7). The median microbiopsy volume was slightly higher for the trachea than for the lung epithelium (Figure S7A).

**Figure 5:**
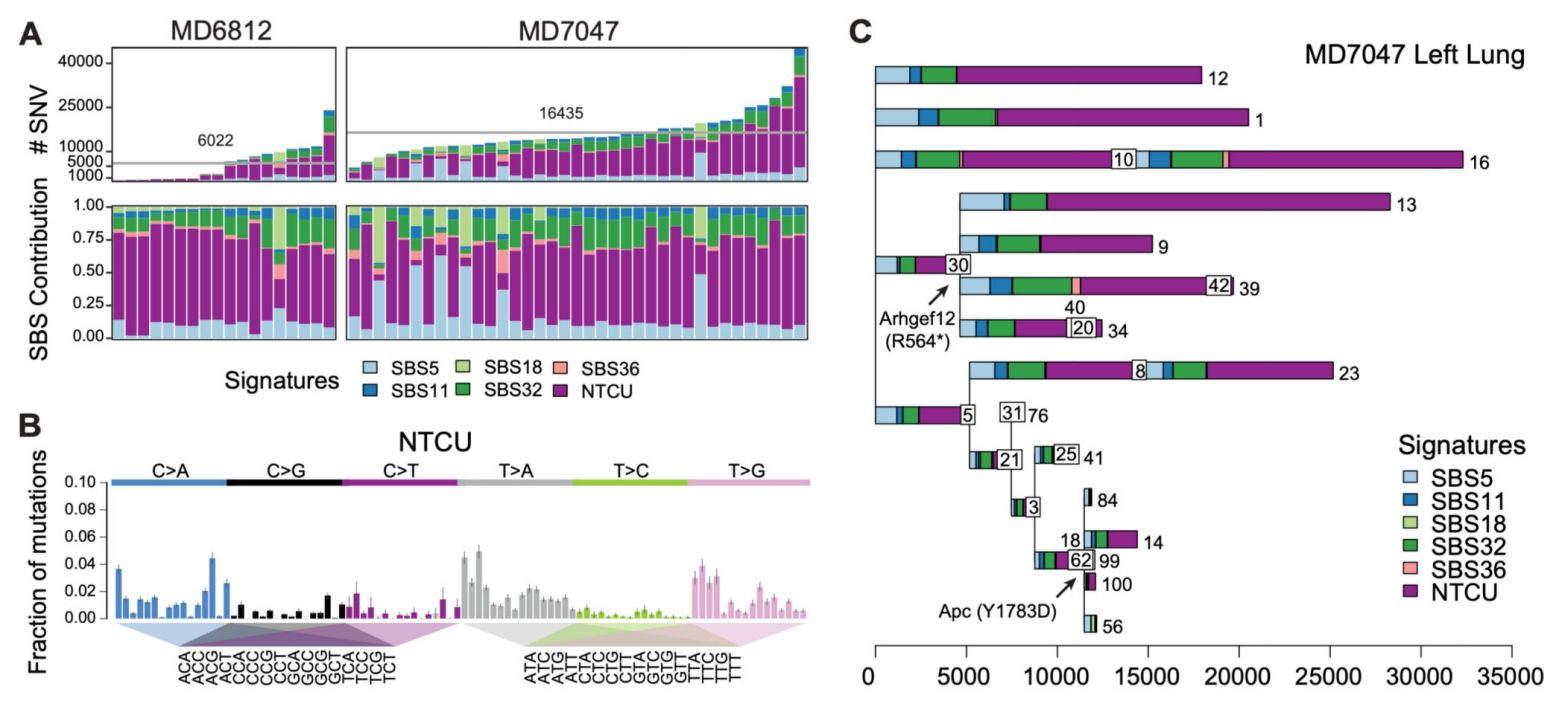
Mutagenic effect of NTCU on basal cells in the airway. **(A)** Burden of single nucleotide variants (SNVs) and single base substitutions (SBSs) signatures, across clones detected in both NTCU-treated mice. Stacked bar plots showing the proportional contribution of each mutational signature to the SNVs, with purple highlighting the NTCU signature. The grey line highlights the average mutation burden across clones. **(B)** Trinucleotide context spectrum of the NTCU signature. **(C)** Phylogenetic tree for samples and clones located on the left lung of mouse MD7047. Clones are highlighted with individual numbers, and mutations colored according to the respective mutational signature contributing to each branch. The boxed numbers represent progenitors of the clones branching of the respective box. Where boxes overlap, the clone number is displayed above the box. Selected mutations in driver genes are annotated on some branches including the amino acid change.

Initially, we used our WGS data to investigate the burden of single base pair substitutions (SBS) as well as indels across both mice using bespoke computational workflows. An individual microbiopsy can contain cells from multiple clonal populations, manifesting as clusters of mutations found at similar variant allele fractions (VAFs). Given the heterozygosity of somatic mutations, a perfectly clonal microbiopsy where all cells derived from a recent common ancestor will have a VAF distribution centered around 0.5. Hence, an increase in the number of sampled clones will result in a shift of this center towards lower VAFs. To evaluate the abundance of clones across microbiopsies, we leveraged a N-dimensional Dirichlet process (39). Given the high number of mutations detected and to facilitate comparisons, we separated microbiopsies according to their location in the left or right lung. This allowed us to compare the burden of SBS and indels per clone across distinct anatomical regions (Figure 5A; Figure S7C; Data S8) (40). Since the ability to detect somatic mutations assigned to a clone is highly dependent on the sequencing depth of the underlying microbiopsy, we performed a logistic regression to adjust the burden per clone according to its predicted sensitivity (Figure S7B). Our results demonstrated considerable variability in burden of single nucleotide variants (SNVs), with a mean of 6022 and 16345 single-base substitutions for MD6812 and MD7047, respectively. In contrast, the average burden of double-base substitutions and indels was on par between both mice (mean=17 and 26 DBS; 15 and 12 indels, respectively).

We used the underlying substitutions assigned to each clone to perform mutational signature analysis. Mutational signatures for each clone were extracted through a Bayesian hierarchical Dirichlet process, assessing their similarity to the bespoke reference signatures from COSMIC (Figure 5A, Figure S7D,E). We found prevalent mutational signatures related to clock-like processes accumulating linearly with age, most importantly SBS5 (Figure 5A) (41). Well-known chemotherapy signatures including SBS11, SBS32 and SBS36 were also common in our dataset. However, the most dominant signature in the dataset was not listed in COSMIC (v3.2). This signature is characterized by T>A and T>G substitutions at ATN sites (Figure 5B), sharing features with previously described signatures of alkylating agents and therefore directly relating to NTCU treatment. We used SigProfiler as an independent approach to validate this NTCU signature (Figure S7E) (42). The signature was present in clones in both trachea and lung and accounted for a total of 36% of all mutations in the whole-genome data, as well as up to 85% of the mutations in individual clones (Figure 5A). Across the dataset in general, both the double-base substitution and indel mutation spectrum did not show a higher-than-expected burden of alterations, with no obvious NTCU-related mutagenic effect (Figure S7C).

Lastly, we inferred phylogenetic relationships between clones in all microbiopsies using the pigeonhole principle, resulting in a total of 4 phylogenetic trees, with the tip of each representing a clone (Figure 5C, Figure S8A-D). We frequently observed larger clades where individual clones descended from a common ancestor (Figure 5C). Due to the small sample size, a formal analysis for recurrence of coding mutations revealed no significant hits (Figure S8). However, we did observe likely functional mutations in genes known to be mutated in human LUSC among the founder mutations of large clonal expansions. In summary, the genomic landscape of the NTCU-treated airways shows an imprint of mutagen exposure, highlighted by a distinct mutational signature. The clones in this dataset occasionally show a high phylogenetic relationship where a highly mutated common ancestor gives rise to several descendants.

## Non-neutral basal cell competition drives progressive colonization and clonal expansions in the airways

**Figure 6:**
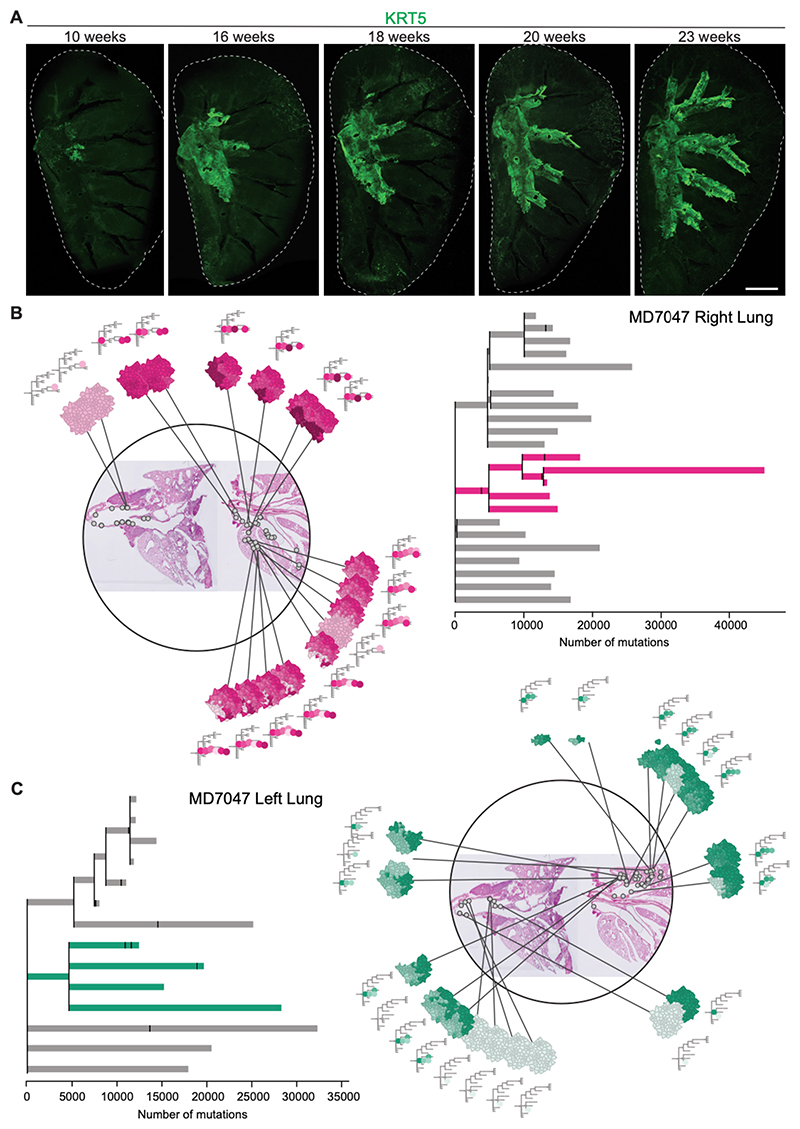
NTCU-driven clonal expansions in the lung. **(A)** 3D projections of whole-mount lungs collected at different time points from NTCU treatment commencement. KRT5 immunostaining was used to visualize basal cells and preinvasive lesions. Scale bar, 2 mm. **(B)** Integrative visualization of the location of a selected clone and respective microbiopsies in the trachea and lung of mouse MD7047. All microbiopsies from the trachea and the right lung containing the magenta clone (lineage) are shown as grey circles within the histological images. The trachea is seen in the tissue section on the left side of the image; the bronchial tree is displayed in the section to the right. The phylogenetic tree depicted on the right-hand side is scaled according to the number of mutations per clone. The magenta ancestor and all subclones related to this clade are highlighted. The small tree schematic surrounding the histological image is equivalent in structure, but not scaled to the mutation burden of each clone. Each dot on the small tree represents a clone and branching point within the phylogeny. **(C)** Equivalent to (B) but for all samples from the trachea and left lung of mouse MD7047. All samples containing the green clone (lineage) are depicted. A complete interactive visualization can be found in the supplementary data (Data S9-S10).

We next assessed the influence of NTCU mutagenicity on the clonal dynamics across the intrapulmonary airways, combining immunofluorescence imaging, biophysical modelling, as well as genomic and histological information. In addition to the basal cell pool in the pseudostratified epithelium of the murine upper airways (Figure 1E,F) (43), rare small clusters of p63^+^KRT5^+^ cells that expand following influenza-induced airway damage can be found in the distal lung (44–46). To identify the basal cell populations that contribute to the formation of early lung cancer lesions, we examined KRT5 expression in lung whole-mounts at different time points from the start of NTCU treatment (Figure 6A). We found that following carcinogen exposure, KRT5^+^ cells gradually occupy the intrapulmonary airways in a proximal to distal fashion, creating an advancing front. No evident discrete peribronchiolar KRT5^+^ cell clusters were observed. This suggests that squamous lung lesions develop from proximal airway basal cells that expand beyond their niche, progressively colonizing the peripheral airways.

During homeostasis and repair, basal cells have multi-lineage differentiation capacity (6, 9). We therefore investigated whether KRT5 lineage-labeled cells that colonize the intrapulmonary airways produce differentiated progeny. Immunostaining analyses of lung sections from *KRT5-CreER;tdTomato* mice sequentially treated with tamoxifen and NTCU revealed areas along the bronchi and bronchioles lined with a seemingly pseudostratified tdTomato^+^ epithelium. These tdTomato^+^ regions contained basal, secretory, and ciliated cells, as assessed by expression of KRT5, SCGB1A1, and acetylated alpha-Tubulin (ACT), respectively (Figure S9A-C). This indicates that at least a subset of basal cells that expand distally after mutagen treatment remains differentiation-competent, uncovering a mechanism through which highly aberrant basal cells may establish a cancerized field containing the various cell types of a histologically normal epithelium. Expression of KRT13 was found to be enriched at the advancing front of the expanding tdTomato^+^ domain, as well as in squamous lesions (Figure S9D).

To evaluate clonal dynamics as basal cells expand into the intrapulmonary airways, we integrated phylogenetic trees with their anatomical information, mapping clones to the epithelial regions where the relevant microbiopsy had been taken (Figure 6B,C; Figure S10,11; Data S8-S12). Several observations emerged from these analyses. First, when focusing on MD7047, we detected four major lineages distributed over millimeters of pulmonary epithelium (Figure 6B,C; Figure S10; Data S9,S10). Second, all clones detected in the lung were related to the common ancestors, while the proximal airway appeared to be more heterogeneous in clonal composition. Third, the spatial territories occupied by descendants of each major lineage were largely exclusive, although they were spatially close to each other. As such, in the right lung of MD7047 distinct clones colonized different lobes (Figure 6B; Figure S10A). These observations were consistent with the non-neutral theory of clonal expansion applied to the airways, which predicted a reduction in clonal heterogeneity as clones colonized a single airway (Figure S12A,C,D and Supplementary Text). Importantly, these features of the spatial distribution of clones were all replicated in the other lobes of MD7047 as well as in a different individual, MD6812 (Figure 6C; Figure S10B, Figure S11). The dominant lineages did not show a particularly higher number of mutations compared to the other clones, although, as mentioned before, they sometimes presented driver mutations (Figure 5C, Figure S8). Taken together, our data demonstrate that the lungs of NTCU-treated mice are dominated by a small number of lineages. The exclusivity of territories occupied by these clones suggests that individual lobes are uniquely colonized by populations derived from a common ancestor. While we can only occasionally find contributions of these clones in the extrapulmonary bronchi, our whole-mount immunofluorescence indicates that the common ancestors of these clones may arise in the upper airways, subsequently migrating distally.

## Clonal relatedness of human preinvasive lung lesions across distinct anatomical sites

**Figure 7:**
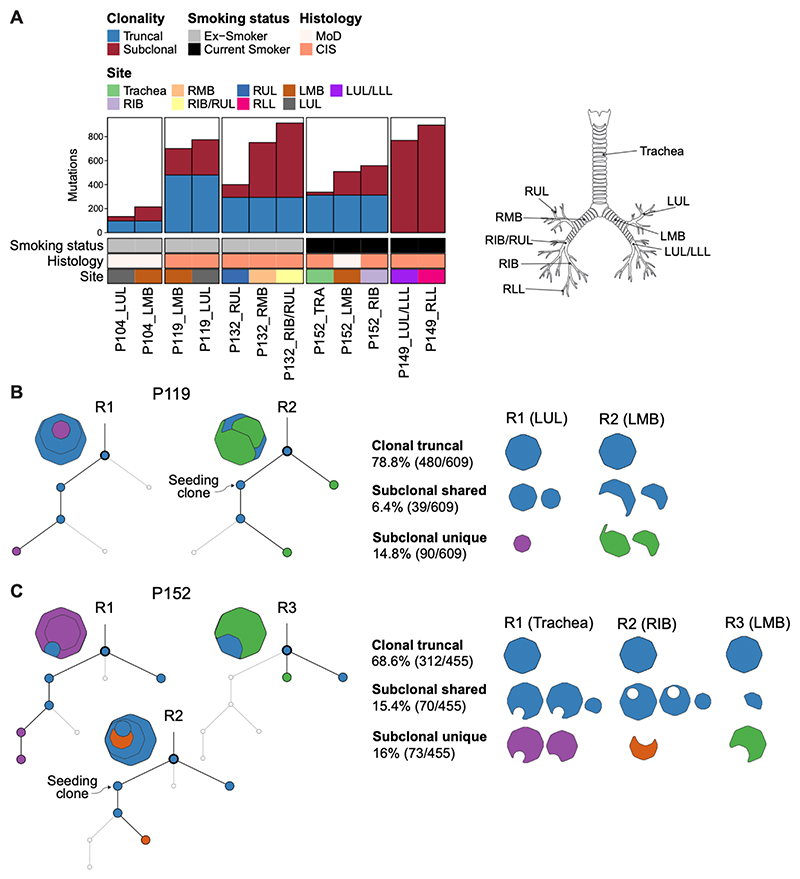
Clonal relatedness between anatomically distinct human preinvasive lung lesions. **(A)** Summary of preinvasive samples and patient characteristics used for assessment of clonality. Schematic shows the anatomical location of biopsy sites. MoD, moderate dysplasia; CIS, carcinoma in situ; RUL, right upper lobe; RMB, right main bronchus; RIB, right intermediate bronchus; RLL, right lower lobe; LUL, left upper lobe; LMB, left main bronchus; LLL, left lower lobe. **(B)** Phylogenetic tree based on somatic mutations illustrating the clonal relationships and evolutionary history of two indolent lesions present in an ex-smoker patient. **(C)** Phylogenetic tree depicting clonal relationships between indolent (R1 & R3) and progressive (R2) lesions in a current smoker. In B-C shared clusters across two or more anatomical sites are colored in blue, while unique and site-specific clusters are colored in purple, orange, or green. Clonal relationships between regions and seeding clones are shown.

Work by Franklin and colleagues proposed the presence of clonally related preinvasive lesions in widely dispersed sites of the human bronchial epithelium (47), supporting the presence of a cancerized field. Subsequent longitudinal analyses by our team suggested that migration of lesion precursor cells contributes to formation of the field along the tracheobronchial tree (48). Our findings in the NTCU model agree with this notion. To explore this further, we used multi-site sequencing to investigate clonal relationships between anatomically distinct preinvasive lesions in five patients recruited to the University College London Hospital Surveillance Study (Data S4). Biopsies with histology ranging from moderate dysplasia to CIS were enriched for epithelial tissue using LCM and subjected to whole-exome sequencing (WES) to a median depth of 431x. We analyzed 12 regions across these donors and found evidence of clonal relatedness between spatially distinct preinvasive airway lesions in 4 out of 5 individuals (Figure 7A; Figure S13). In one patient, P152, truncal events spanned lesions in the trachea and both the left and right bronchi (Figure 7A), indicating that lesion precursor cells can spread bilaterally.

To elucidate the dynamics of somatic events across different sites, we constructed phylogenetic trees for each patient using CONIPHER (49), which integrates Dirichlet clustering and copy number error correction of somatic mutations. Within these phylogenetic trees, clusters containing all other clusters were classified as truncal mutations, while the remaining were categorized as subclonal mutations (Figure 7B,C; Figure S14A,B; Data S13). Although analysis of selection of lung cancer gene mutations (Data S5) was underpowered due to the patient cohort size, mutations in *TP53* were identified as the most significantly selected truncal event (*q*=0.015), present in 100% of clonally related sites (Figure S14C). The proportion of clonal truncal mutations ranged from 26.4% to 78.8% in patients with clonally related lesions, whereas shared subclonal mutations were observed at frequencies of 6.4% to 15.4% (Figure 7B,C; Figure S14A,B). The identified clonal diversity between regions indicates that cells accumulate additional mutations over time as they migrate between sites. One patient, P149, presented two anatomically separate lesions (LUL/LLL and RLL) arising independently, each harboring distinct *TP53* mutations (Figure S14D).

Utilizing the mutation clonality information derived from the phylogenetic analysis, we investigated the dynamics of clonal and subclonal fractions of the top six SBS signatures observed in CIS and LUSC (4, 50), namely SBS1, SBS2, SBS4, SBS5, SBS13, and SBS92. Consistent with previous studies (4), tobacco-associated signatures (SBS4 and SBS92) were highly prevalent in preinvasive lesions, contributing between 12% and 45% of all mutations (Figure S15A), similar to what has been reported for LUSC (50). On average, SBS4 was more enriched in truncal mutations compared to subclonal mutations, suggesting that DNA damage caused by cigarette smoke drives early clonal expansion and disease progression (Figure S15A, B). In contrast, APOBEC signatures (SBS2 and SBS13) were more enriched in subclonal mutations (Figure S15A, B), indicating that mutations resulting from APOBEC activity are likely later events in lung squamous cell carcinogenesis.

## Discussion

We have tracked the development of field cancerization in the airway epithelium and demonstrated that preinvasive lung squamous lesions originate from basal cells. Using a carcinogen-induced murine model of LUSC, we show that carcinogen exposure shifts epithelial cell fate and drives non-neutral competition among basal cells, leading to large clonal expansions of mutant cells along the length of the tracheobronchial epithelium. Preinvasive lesions eventually emerge from a few dominant mutant clones that escape the confines of the tracheal niche and progressively expand to colonize the bronchial tree.

The mutational landscape of the histologically normal human bronchial epithelium suggests that selection of mutant clones starts before the appearance of preinvasive disease (2). Given the inherent limitations of human studies, resolving this process in the context of the airway architecture is challenging. The murine model of LUSC we have used here allowed us to overcome this. The ability to visualize clonal distributions on tissue whole-mounts and to perform extensive sampling across the bronchial tree enabled us to delineate how mutagenic insults shape clonal dynamics, and to understand their transcriptomic and genomic consequences.

We found that carcinogen exposure shifted basal cell fate in the murine upper airways, favoring a *Krt14*^high^ basal cell state. This change was accompanied by an accumulation of a transitional *Krt4/Krt13*^+^ cell population (7) in the basal and suprabasal epithelial cell layers, and decreased presence of secretory and ciliated cells, suggesting impaired progression towards luminal cell fate. We identified increased expression of *KRT4/KRT13* in the human airways of smokers, as well as in preinvasive squamous cell lesions and LUSC. A subpopulation of *Krt13*^+^ cells localized to discrete sites of the tracheal epithelium named ‘hillocks’ has been recently shown to be a source of vitamin A deficiency-induced murine squamous metaplasia (51). Our lineage tracing studies demonstrate that NTCU-driven squamous disease originates from *Krt5*-expressing basal cells. While our basal cell labeling strategy would track *Krt5/Krt13*^+^ hillock basal cells, aberrant basal cell clonal expansions were not restricted to specific tracheal regions, but evident throughout the epithelium of the upper airways, making hillock basal cells solely responsible for the phenotype unlikely. Our cell trajectory analyses both in mouse and human, however, highlight the transition of basal cells into a *Krt4/Krt13*^+^ cell state as an early step during precancerous squamous disease development, suggesting potential shared properties between this cellular population and hillock-derived *Krt13*^+^ cells.

Through multi-site sequencing we demonstrate the presence of clonally related preinvasive lesions across distinct anatomical sites of the lung both in the mouse and humans, indicating that migration of preinvasive lesion precursor cells across the bronchial tree contributes to field cancerization in the airways, as previously postulated (48). In the NTCU model, mutant cells progressively mobilize from the major airways into the bronchioles, areas that are normally devoid of basal cells. These migratory cells produce both differentiated luminal cells in their new environment—resulting in ectopic areas of pseudostratified epithelium—and precancerous lesions. In the human airways, it appears precancerous basal cells undergo similar migration and expansion, producing distinct but clonally related lesions. Mobilization of subpopulations of airway epithelial cells in the intrapulmonary airways has been observed in mice following severe injury (52, 53). While some of these cell subpopulations have been shown to activate expression of KRT5, they were mostly found to emerge from distal epithelial cell populations, and only a minority was reported to derive from pre-existing basal cells (52, 54). Further studies are required to delineate the mechanisms underlying the migration of basal cell-derived lineages, and to understand the signals that restrict basal cell domains in normal homeostatic conditions.

Taken together, our work identifies the disruption of basal cell homeostasis as a key cellular event underlying the initiation of lung squamous carcinogenesis.

## Materials and methods summary

### Mouse models

Work involving the use of animal models was approved by the UCL Animal Welfare Ethical Review Body and performed in accordance with the UK Home Office procedural and ethical guidelines. FVB/N mice were purchased from Charles River UK. The *Tg(KRT5-CreER), Scgb1a1-CreER*^™^, *R26R-Confetti* and *R26R-CAG-LSL-tdTomato* lines have been described previously (9, 11, 55–57). Transgenic lines were backcrossed to FVB/N for at least 2 generations before producing experimental cohorts. All mice were maintained in individually ventilated cages with access to food and water *ad libitum*. For experiments involving the use of transgenic animals, littermates were randomly distributed between treatment and control groups.

### NTCU-induced lung carcinogenesis

N-nitroso-tris-(2-chloroethyl)urea (NTCU, Santa Cruz Biotechnology sc-212265) was administered to 6-week-old female mice as previously described (20). Briefly, 75 μL of 13 mM NTCU in acetone were applied onto the shaved back of each mouse twice weekly for 12 weeks. Treatment was followed by a monitoring time of up to 12 weeks. Where indicated, 5-ethynyl-2’-deoxyuridine (EdU, ThermoFisher A10044 or Merck 900584) in sterile phosphate buffered saline (PBS) was administered intraperitoneally at 50 μg/g of body weight to label dividing cells. At the experimental end point, mice were terminally anesthetized and transcardially perfused with PBS prior to tissue collection.

### Human sample collection for single-cell RNA sequencing

Ethical approval for patient sample collection was obtained through the UCL/UCLH Local Ethics Committee under the study REC reference 18/SC/0514.

Patients were recruited via screening of ENT operating lists or bronchoscopy lists; those aged 50-75 years old without active cancer or infection were included. Three never-smokers and three current smokers were identified; all patients provided informed, written consent. Tracheal brushings were obtained during routine diagnostic or therapeutic microlaryngoscopy under general anesthesia or flexible bronchoscopy under sedation, placed immediately into transport media (alpha-MEM containing 1X penicillin-streptomycin (Gibco, 15070), 250 ng/ml amphotericin B (Thermo Fisher Scientific, 10746254) and 10 ng/mL gentamicin (Gibco, 15710)) and transported on wet ice directly to the laboratory for processing. The tracheal brushes were processed into single cell suspension according to previously published protocol by Worlock et al (58). Trypan Blue was used to assess cell count and viability prior to resuspending in HBSS/BSA 0.05% at 1000 cells/μL. 10X single-cell gene expression libraries were prepared at the CRUK City of London Single Cell Genomics Facility using 5’ reagents for 5,000 targeted cell recovery.

### Human sample collection for whole-exome sequencing

Patient samples were collected under the study REC reference 01/0148, which was approved by the UCL/UCLH Local Ethics Committee.

Patients were recruited to the University College London Hospitals preinvasive surveillance study, where they undergo periodic autofluorescence bronchoscopy (59). At bronchoscopy, biopsies of regions identified as abnormal to autofluorescence were taken, embedded in OCT and frozen on dry ice. An additional biopsy was taken for histopathological assessment, as well as a blood sample, which was used as germline control. Fresh frozen biopsies were serially cryosectioned at 7-10 μm thickness and mounted onto MembraneSlide 1.0 PEN slides. Every 12 sections, a reference section was collected and stained with hematoxylin and eosin (H&E) to confirm histology and locate areas of preinvasive disease. LCM of the region of interest was performed on the PALM Microbeam system (Carl Zeiss MicroImaging, Munich, Germany). DNA from the micro-dissected epithelium and from 1.5 mL of whole blood was extracted using the QIAGEN QIAmp DNA micro kit (Crawley, UK), according to the manufacturer’s instructions. DNA yield was increased by using soluble carrier RNA in the DNA extraction process and final DNA concentration was calculated using the Qubit dsDNA High-Sensitivity assay (Life Technologies, Paisley, UK). Only samples with A260/280 absorbance ratio readings of 1.7-1.9 were included.

## Computational Methods

### Sub-clustering of mouse tracheal epithelial cells

Murine epithelial cells were sub-clustered using the Louvain algorithm with a resolution of 0.6 to resolve finer cellular distinctions. Cell type annotations of these sub-clusters were informed by the expression of top cluster-specific markers idented by *FindAllMarkers* function (Data S3) and cross-referenced with consensus genes (Data S2) from the literature. To distinguish between different basal cell phenotypes, we assessed their unique transcriptional profiles by leveraging top markers identified in our data and previously published studies. Marker gene dotplots and UMAP visualizations were generated to provide robust validation of cell type assignments and distinguish basal cell subtypes.

### Compositional analysis of murine airway with scCODA

To evaluate whether the abundance of any of the identified epithelial cell types changed by NTCU treatment, we used scCODA, a Bayesian model to assess compositional changes in pre-defined clusters from single-cell data (https://sccoda.readthedocs.io/en/latest/) (24). Using a hierarchical Dirichlet-Multinomal model, scCODA accounts for uncertainty in cell-type proportions as well as the negative correlative bias across cell-type proportions in relation to a reference cell type. Ciliated cells were used as reference for our analysis, however, it should be noted that the results did not change substantially when allowing scCODA to automatically determine the reference cell type. In addition to the reference cell type, we specified the treatment condition and the individual mouse as covariates. The remaining analysis was implemented as described in the single-cell best practice vignette (https://www.sc-best-practices.org/conditions/compositional.html).

### Pseudotime trajectory analysis for murine samples

We employed Monocle2 (2.24.0) (28) for pseudotime analysis of basal, Krt4/Krt13^+^ and secretory cells. A single-cell trajectory was constructed using the Discriminative Dimensionality Reduction with Trees (DDRTree) algorithm, employing the top 400 significantly differentially expressed genes among the selected epithelial cell types. Cells were ordered along the trajectory with the state containing proliferative basal cells set as time zero, and pseudotime was calculated accordingly. To ensure clarity in trajectory dynamics visualization, cell numbers in each group were downsampled by 10%. Trajectory plots were generated using the *plot_cell_trajectory* function. The log2 fold change for cell abundance was computed for each cell type on each cell state, with sample size adjustments factored in using R.

### Integration of human single-cell RNA-seq data with publicly available datasets

Our tracheal scRNA-seq data (n = 6) was integrated with publicly available tracheal datasets from current- and/or non-smokers generated by Goldfarbmuren *et al*., 2020 (29) (GSE134174, n = 12), and Deprez *et al*., 2020 (10) (EGAS00001004082; n = 9). Integration and data processing were conducted using Seurat v5.0.1. Expression values for each cell were then normalized using the global-scaling normalization method *LogNormalize*. Principal component analysis (PCA) was performed on the top 2000 highly variable genes, excluding mitochondrial and ribosomal genes. The optimal number of principal components (PCs) for further analysis was selected based on a scree plot. To address batch effects across datasets and donors, we applied the Harmony algorithm with theta 1 to compute a batch-corrected UMAP. Louvain clustering was then performed using the *FindClusters* function with a resolution of 0.6, which was identified as the best resolution for accurately separating refined cell types. Differential cell markers within each cluster were identified using the *FindAllMarkers* function, with a threshold of 25% minimum expression percentage, and minimum log fold change of 0.25 (Data S6). Top marker genes for each cluster were visualized using heatmaps to confirm specificity.

To annotate clusters, the top identified marker genes for each cluster were cross-referenced with consensus marker genes reported across multiple studies (Data S5). UMAP visualizations and heatmaps were generated to validate these annotations. The cluster-specific markers and their correspondence with published signatures ensured robust identification of cell types across datasets.

### Differential cell abundance analysis of human epithelial cell types with MiloR

To investigate differential abundance of tracheal epithelial cell types between non- and current-smokers we used MiloR v 1.4.0 (34). This approach enabled us to detect changes across a dynamic cell population, where cells may be transitioning through cell states, by analyzing local neighborhood information. The analysis involved: creating a Milo object from the batch-corrected graph of the integrated datasets, building the graph with parameters k = 30 and d = 30, and generating neighborhoods using *makeNhoods* with prop = 0.1, k = 30, d = 30, and refined = TRUE. Cells within neighborhoods were counted using the *countCells* function at the sample level and neighborhood distances calculated using *calcNhoodDistance* with d = 30. Differential abundance was assessed by applying a SpatialFDR threshold of ≤ 0.1 in any single neighborhood.

### Single-base-substitution calling in mouse WGS

Single nucleotide variants were called using the Cancer Variants through Expectation Maximization (CaVEMan) algorithm (60) with copy-number options of major copy number 5, minor copy number 2 and normal contamination 0.1. In cases where samples had a CNA, the ASCAT results were incorporated in the variant calling. In addition to the default ‘PASS’ filter, we removed variants with a median alignment score (ASMD) < 120 and those with a clipping index (CLPM) > 0, to remove mapping artefacts. Subsequently, for every mutation identified in any sample from each patient, we counted the number of mutant and wild-type reads using vafCorrect (https://github.com/cancerit/vafCorrect). Further filters described below were applied to identify true somatic mutations and separate them from either germline variants or recurrent sequencing errors.

### Identification of clones through SNV clusters in mouse WGS

A nonparametric Bayesian hierarchical Dirichlet process (HDP) was implemented to cluster SNVs with similar variant allele frequencies (VAFs) that were called across multiple microdissections for each patient biopsy as described previously (39). This N-dimensional Dirichlet process (NDP) clustering approach was run with 5,000 burn-in iterations, followed by 5,000 posterior Gibbs sampling iterations that were used for clustering. In principle, there is no requirement to pre-specify the number of clusters, making this process flexible for all datasets. In this way, the number of SNV clusters are permitted to vary throughout the sampling chain. Only SNV clusters comprising a minimum of 50 unique mutations were kept for downstream analysis. Input to this algorithm included per-patient data tables consisting of the coverage and counts of each called variant per microdissection.

### Inference of phylogenetic trees

The statistical pigeonhole principle was applied to infer phylogenetic clonal relationships between per-patient SNV clusters identified by the NDP algorithm as highlighted previously (39). Thereby, each evaluated cluster is represented as a branch of a phylogenetic tree. A given cluster is considered to have strong evidence of being nested within another (that is, sub-clonal relationship) if the fraction of cells carrying the cluster of mutations is lower in all member microdissections relative to the fraction of cells containing another cluster of mutations within the same microdissections, in which the sum of their respective mutant cell fractions (CFs) is also >100%. Otherwise, if the sum of the pairwise mutant CFs is ≤ 100%, only weak evidence of nesting exists. In cases in which only some microdissections have lower CFs of a given SNV cluster relative to another, the clusters are interpreted to be independent and not nested within one another. Here, only clusters with a median VAF ≥ 0.1 are analyzed.

### Interactive visualizations using MapScape

To combine genomic and histological information, a custom R script based on the MapScape package was developed (https://github.com/shahcompbio/mapscape). A histology image, the pixel location of the samples, as well as the information of clonal prevalence generated by the NDP were leveraged as input. The phylogenetic tree, represented as a table containing the clone structure, the phylo object as well as the mutations assigned to each branch of the tree were provided as inputs as well. The resulting output was saved as html to enable interactive exploration of the data.

### Somatic SNV calling in human samples

Lesion reads were compared to the germline DNA (blood) to identify somatic single nucleotide variants (SNV) using MuTect2 v4.2.0 (61). MuTect2 calls were filtered for ‘PASS’. Additional filtering was performed to minimize false-positive variant calls using FilterMutectCalls (flags: --min-median-base-quality 20; --min-median-mapping-quality 30; --max-events-in-region 2; - -max-alt-allele-count 1).

VarScan2 somatic (v2.3.9) (62) was also used to call the somatic variants. VarScan2 output from SAMtools mpileup (minimum mapping quality = 20) was used to identify somatic variants between lesion and matched germline samples. The following filters were applied to VarScan2: lesion depth ≥ 30, germline depth ≥30, Variant Allele Fraction (VAF) ≥ 0.03, with a somatic p-value ≤ 0.01. A variant allele frequency ≥ 0.05 applied if only called in VarScan2. Additionally, a blacklist filter was applied to some genomic location of the variant. Blacklisted regions include regions identified as problematic regions of the genome in the Encode project (63).

The somatic callers were annotated against database sources including COSMIC (v.75, https://cancer.sanger.ac.uk/cosmic) (64), RefSeq and other in silico prediction tools using ANNOVAR v1.0.0 (65). As a preparation step for subclonality and phylogeny analysis and to ensure comprehensive detection and consistent monitoring of somatic variants, MuTect2 v4.2.0 (61) force calling was performed on all somatic variants that passed the quality control on multi-region samples. This approach allowed us to accurately track the evolution of precancerous cells by ensuring variant calling at these sites across all samples for each patient.

### Somatic Copy Number Alteration (CNA) detection in human WES

Allele-specific copy number analysis was conducted using ASCAT v3.1.2 (66), as per the authors’ recommendations for WES data (https://github.com/VanLoo-lab/ascat/tree/master/ReferenceFiles/WES/). In summary, allele counts were obtained using alleleCounter v4.3.0 (https://github.com/cancerit/alleleCount), which quantified the number of reads supporting each allele at SNP sites of G1000_loci_hg19. These counts were then converted into logR and B-allele frequency (BAF) values using the updated ascat.prepareTargetedSeq() function, which employs a probabilistic method to infer genotypes based on read counts. The input data for ASCAT was subjected to GC correction, using a wave-pattern GC correction calculated with the ASCAT-provided scripts. The segmentation output was generated using the ascat.runAscat() function with gamma set to 1. For subclonality analysis, only copy number segmentations from autosomes and samples with a purity greater than 10% were included.

### Subclonality and generation of phylogenetic trees

To infer the clonality and diversity of preinvasive lesions, somatic SNVs with a similar cellular prevalence were clustered using CONIPHER v1.0. (49). Briefly, this process was conducted in three steps for each patient: (I) estimation of the proportion of preinvasive cells in which a given mutation is present in each sample, using the VAF corrected for local copy number and purity; (II) identification of mutation clusters; (III) reconstruction of phylogenetic tree by correcting for complex evolutionary events like mutation losses, and removing biologically improbable mutation clusters. The minimum number of mutations per cluster was set to 5. The clonality of mutational clusters was inferred based on the cell fraction estimated from the phylogeny. Phylogenetic trees were visualized using the R package ggplot2. The subclonal structure for each sample was illustrated using cell fractions and nesting structure, determined by the phylogenetic tree, with the R cloneMap package (https://github.com/amf71/cloneMap) (67).

## Supplementary Material

Data S1

Data S2

Data S3

Data S4

Data S5

Data S6

Data S7

Data S8

Data S9

Data S10

Data S11

Data S12

Data S13

Supplementary Materials

Supplementary References

## Data Availability

Sequencing data are available on ENA (murine WGS: ERP128764; murine scRNA-seq: ERP136782), GEO (human scRNA-seq: GSE276610) and HTAN (human WES: dbGaP phs002371). Data objects and a curated list of murine variant calls are provided on Dryad (40). Computational methods provide a summary of the procedures implemented in various custom-made R, Python and Bash scripts. These scripts contain the commands run for the analyses highlighted in this publication. To sustain reproducibility, the code is publicly available on Zenodo (68). Here we also included the void size data and MATLAB (The MathWorks Inc., Natick, Massachusetts, USA) scripts to run the simulations and analyses of the neutral and non-neutral models.
