# Supplementary material for: Aberrant basal cell clonal dynamics shape early lung carcinogenesis
Source: Science. Author manuscript; Available in PMC 2025 Jun 23. (PMC7617789; doi:10.1126/science.ads9145)
Supplement: Data S9 [file EMS206506-supplement-Data_S9.html]

Mapscape Generation


# Mapscape Generation

#### Moritz Przybilla

#### 31 January, 2025

#### Filter to remove samples withough any clone > 0.01

(if low\_prev = TRUE, this step is skipped)

```
### If only high-prevalence clones being displayed, remove microbiopsies without any clones >0.01 (otherwise mapscape fails)
if (low_prev == FALSE) {
  print("removing low prevelence (<0.01) samples from display")

  max_prev <- prev_tbl_filt %>%
    dplyr::group_by(sample_id) %>%
    dplyr::summarise(max_prev = max(clonal_prev))
  high_prev_samples <- max_prev %>%
    dplyr::filter(max_prev >= 0.01) %>%
    dplyr::select(sample_id)
  removed_samps <- max_prev %>%
    dplyr::filter(max_prev < 0.01) %>%
    dplyr::select(sample_id)
  removed_samps <- removed_samps$sample_id
  high_prev_samples <- high_prev_samples$sample_id
  print(removed_samps)
  prev_tbl_filt <- prev_tbl_filt %>%
    dplyr::filter(sample_id %in% high_prev_samples)
  location_tbl <- location_tbl %>%
    dplyr::filter(sample_id %in% high_prev_samples)
  
  # filter mut table to only inclde samples/clones present
  present_clones <- unique(prev_tbl_filt$clone_id)
  if (exists("mut_tbl_final")) {
      mut_tbl_final <- mut_tbl_final %>%
    dplyr::filter(sample_id %in% high_prev_samples,
                  clone_id %in% present_clones) 
  }
} else {
  print("Low prevelence clones displayed, entire tree included")
}
```

```
## [1] "removing low prevelence (<0.01) samples from display"
## factor(0)
## 61 Levels: d_0003 d_0004 d_0015 d_0016 d_0033 d_0034 d_0065 d_0066 ... d_0161
```

### Display cluster diagram

```
cluster_diagram_file <- paste0(ndp_dir, "/Cluster_and_spectrum_plots.pdf")
```

### Display per-cluster mutation counts

```
mut_counts %>%
  kable %>%
  kable_styling("striped", full_width = F) %>%
  scroll_box(width = "300px", height = "500px")
```

| Cluster ID | Mutations Assigned |
| --- | --- |
| Cl.1 | 17551 |
| Cl.3 | 1288 |
| Cl.5 | 5167 |
| Cl.8 | 9413 |
| Cl.9 | 9377 |
| Cl.10 | 12969 |
| Cl.12 | 11958 |
| Cl.13 | 8317 |
| Cl.14 | 2795 |
| Cl.16 | 14745 |
| Cl.18 | 2610 |
| Cl.20 | 258 |
| Cl.21 | 2298 |
| Cl.23 | 8744 |
| Cl.25 | 1844 |
| Cl.30 | 4636 |
| Cl.31 | 160 |
| Cl.34 | 835 |
| Cl.39 | 598 |
| Cl.40 | 6643 |
| Cl.41 | 91 |
| Cl.42 | 14330 |
| Cl.56 | 670 |
| Cl.62 | 40 |
| Cl.76 | 348 |
| Cl.84 | 392 |
| Cl.99 | 65 |
| Cl.100 | 521 |

### Display SigFit Results

```
#SigFit_reconstruction_file <- paste0(SigFit.dir, substr(sample_name, 1,7), "_SigFit_reconstruction.pdf")

#<object data=`r SigFit_reconstruction_file` width="1200px" height="1200px">
#    <embed src=`r SigFit_reconstruction_file`>
#    </embed>
#</object>
```

#### Display Large Versions of Static Trees

### Generate mapscape

```
if (exists("mut.file")) {
  print("mutation table found")
  mapscape(clonal_prev = prev_tbl_filt, 
         tree_edges = edge_tbl, 
         sample_locations = location_tbl,
         img_ref = image.file,
         static_tree_image = static_tree_file,
         width = 1500,
         height = 1300,
         show_low_prev_gtypes = low_prev,
         phylogeny_title = sample_name,
         anatomy_title = pat_age,
         clone_colours = color_tbl,
         mutations = mut_tbl_final)
} else {
  print("no mutation table found")
  mapscape(clonal_prev = prev_tbl_filt, 
           tree_edges = edge_tbl, 
           sample_locations = location_tbl,
           img_ref = image.file,
           static_tree_image = static_tree_file,
           width = 1500,
           height = 1300,
           show_low_prev_gtypes = low_prev,
           phylogeny_title = sample_name,
           clone_colours = color_tbl)
}
```

```
## [1] "mutation table found"
## [1] "[WARNING] Low prevalence genotypes will not be shown in the view. To show them, set show_low_prev_gtypes parameter to TRUE."
```
