# Supplementary material for: Aberrant basal cell clonal dynamics shape early lung carcinogenesis
Source: Science. Author manuscript; Available in PMC 2025 Jun 23. (PMC7617789; doi:10.1126/science.ads9145)
Supplement: Supplementary Materials [file EMS206506-supplement-Supplementary_Materials.pdf]

Materials and Methods

Tamoxifen administration for lineage-tracing studies

A 20 mg/mL tamoxifen (Sigma T5648) stock solution was prepared in 10% ethanol/corn oil, and administered via oral gavage at 200 µg/g of body weight. Administration regimes used for individual experiments are indicated in the main text and/or figure legends.

Histology and immunostaining

Lungs were insufflated with 4% paraformaldehyde (PFA)/PBS before collection. For whole-mount staining of samples expressing fluorescent reporters, tissues were fixed in ice-cold PFA for 2h, rinsed in PBS and transferred to PBS containing 0.05% ProClin 300 (Sigma 48912-U). All other samples were fixed overnight at 4°C before further processing.

Paraffin-embedded tissues were sectioned at 4 µm and dewaxed on an autostainer (Tissue-Tek DRS, Sakura). Heat-mediated antigen retrieval was performed in a microwave oven. Sections were blocked in 5% normal donkey serum/3% bovine serum albumin (BSA)/0.1% Triton X-100/0.05% ProClin 300/PBS for 1-2h at room temperature. When using primary antibodies raised in mouse on mouse tissue, sections were incubated with mouse on mouse blocking reagent (Vector Laboratories MKB-2213-1) 1h at room temperature or overnight at 4°C to block endogenous immunoglobulins. Primary antibodies (Table S1) were applied in blocking solution overnight at 4°C. Sections were washed thrice with 0.1% Triton X-100/PBS (PBST) and incubated with Alexa Fluor- or DyLight-conjugated secondary antibodies (ThermoFisher or Jackson ImmunoResearch) overnight at 4°C. Following three washes with PBST, nuclei were counterstained with 4', 6-diamidino-2-phenylindole (DAPI). Sections were washed twice in PBS before mounting with Fluoromount G (SouthernBiotech 0100-01).

For immunohistochemistry (IHC) staining, sections were incubated with 3% H_2O_2 (ThermoFisher 426001000) for 20 minutes at room temperature after antigen-retrieval, then washed thrice with PBS and blocked with 2.5% normal horse serum (Vector Laboratories MP-7401) for 3h at room temperature. Following primary antibody incubation overnight at 4°C and three washes with PBST, sections were incubated in ImmPRESS polymer reagent (Vector Laboratories MP-7401) for 1h at room temperature and then detected with NovaRed substrate kit (Vector Laboratories SK-4805). IHC and H&E (Tissue-Tek DRS, Sakura) stained sections were imaged using a S360 Nanozoomer (Hamamatsu).

Whole-mount immunofluorescence

The trachea and mainstem bronchi were separated from the lungs and extra-tracheal tissues removed under a dissection microscope (Leica Stereozoom Si9). For lung whole-mounts, the left lobe was microdissected to expose the bronchial tree. Tissues were blocked in 5% normal donkey serum/2.5% BSA/0.5% Triton X-100/4% dimethyl sulfoxide (DMSO)/0.05% ProClin 300/PBS. Samples were incubated with primary antibodies diluted in blocking solution for 48-72h at 4°C with gentle rocking. After 3 washes in PBST at room temperature, Alexa Fluor-conjugated secondary antibodies (ThermoFisher or Jackson ImmunoResearch) were applied in blocking solution for 24-48h at 4°C with gentle rocking. Tissues were washed in PBST and, where indicated, nuclei counterstained with DAPI. Following further washes in PBST and PBS, samples were mounted in RapiClear 1.52 (SUNJin lab RC152001). Prior to mounting, trachea samples were cut either along the flanks to obtain a ventral and a dorsal half, or longitudinally through the ventral midline to expose all the epithelial surface.

EdU staining was performed after primary antibody washes, using a Click-iT Plus EdU Alexa Fluor 488 Imaging Kit (ThermoFisher C10637) and following the manufacturer's recommendations. Confocal images were acquired on a Zeiss LSM 880. Images were processed and analyzed using Fiji.

Mosaic analysis

As labeling in the trachea was not clonal, evidenced by the merger of large labeled regions, we focused our attention in the organization of the clusters of unlabeled cells or voids. For this, we first applied a Gaussian filter (radius 5 pixels) in order to smoothen inhomogeneities and applied an intensity threshold to identify labeled and unlabeled regions. Using the DAPI channel as a mask, we identified connected voids and recorded their area in pixels. We then normalized the areas by the average area of a cell, of about 388± 84(SD) pixels (equating to a radius of 8.0 ± 3.8(SD) µm) for the control and 370+-65 (SD) pixels (equating to a radius of 7.8 ± 3.3(SD) µm) for the NTCU-treated samples. In the analysis, we focused on the proliferative population, considering holes with size of three cells or more. With this information we could plot the cumulative distribution of void sizes, which provided insight into the dynamics of the tissue.

Preparation of murine cells for single-cell RNA sequencing

Terminally anesthetized mice were transcardially perfused with sterile ice-cold PBS prior to tissue collection. Tissues were placed in ice-cold PBS, the trachea and mainstem bronchi were separated from the intrapulmonary airways at the lung junction and extra-tracheal tissues removed under a dissection microscope. The trachea and mainstem bronchi were transferred to Dispase (Corning 354235) and incubated at 37°C for 40 min. The epithelium was flushed out with ice-cold PBS using a syringe attached to a 25G needle, and collected by centrifugation at 300 x g for 5 min at 4°C. Cell pellets were resuspended in 0.05% Trypsin-EDTA (Gibco 25300104) and incubated at 37°C for 10 min. Trypsin was inactivated by adding 10% fetal bovine serum (FBS) (Gibco 10270-106) in PBS, and a single-cell suspension obtained by resuspending with a wide-bore pipette tip. The cell suspension was filtered through a 40 µm strainer and centrifuged 5 min at 300 x g. Cells were frozen in Recovery Cell Culture Freezing Medium (ThermoFisher 12648010) and stored at -150°C.

For library preparation, cells were thawed in warm 10% FBS in RPMI (Gibco 21875-034), collected by centrifugation at 300 x g, and depleted from dead cells using magnetic-activated cell sorting (MACS; Miltenyi 130-090-101) according to 10X recommendations. Live cells were resuspended in 0.04% BSA (ThermoFisher AM2616) in PBS at 1000 cells/µL. 10X single-cell gene expression libraries were prepared at the CRUK City of London Single Cell Genomics Facility using 5' reagents.

Laser capture microdissection and low-input whole-genome sequencing of murine samples

Following transcardial perfusion with ice-cold PBS, lungs were insufflated with PAXgene tissue FIX (Qiagen 765312) from the proximal end of the trachea, isolated and fixed in 15 mL of PAXgene for 24h at room temperature. Samples were placed in PAXgene tissue stabilizer solution (Qiagen 765512) at -80°C for at least 24h. Tissues were cryoprotected in 30% sucrose/PBS for 24h at 4°C, followed by 1:1 optimal cutting temperature (OCT) compound and 30% sucrose/PBS for further 24h, prior to OCT embedding. 15 µm sections were collected onto polyethylene naphthalate (PEN)-membrane slides (Leica 11505158) and used for laser-

capture microdissection (LCM). Microbiopsies were cut, digested with proteinase K and used as input for low-input whole-genome sequencing (WGS) as previously described (*38*). 150-base-pair paired-end sequencing clusters were generated on the Illumina HiSeqX or Novaseq platform according to Illumina no-PCR library protocols.

Library preparation for human whole-exome sequencing

Library preparation was performed on 30 ng of extracted DNA by Oxford Genomics Centre (University of Oxford). Fragments of interest were captured using the Human Core Exome panel (Twist Bioscience, San Francisco, USA), extended by an additional spike-in panel to include intronic regions linked to fusion events in NSCLC. This design was based on the exome panel used by TRACERx (*69*). Samples were 150 bp paired-end multiplex sequenced on the Novaseq 6000. Whole exome sequencing (WES) data was aligned to the reference human genome (hg19) achieving a median sequencing depth of 431 for the abnormal regions and 415 for the matched germline. Lesions were classified as indolent or progressive, depending on whether they remained unchanged or progressed to LUSC during the time of the study, respectively.

Computational Methods

Quality Control and scRNA-seq data pre-processing for murine samples

The raw base call files from the 10X Chromium sequencer were processed using the Cell Ranger Single-Cell Software Suite (release v7.0, https://support.10xgenomics.com/single-cell-gene-expression) according to the manufacturer's instructions, including the commands "cellranger mkfastq", "cellranger count" and "cellranger multi" for paired gene expression and vdj data. The reads from single-cell RNA-sequencing were aligned to the latest mm10 reference genome implementing a pre-built annotation package downloaded from the 10X Genomics website (refdata-gex-mm10-2020-A and refdata-cellranger-vdj-GRCm38-alts-ensembl-7.0.0 for GEX and VDJ respectively). Several output files including a barcoded binary alignment map (bam) file and a summary csv file are generated. For gene expression data, a filtered feature-barcode matrix folder, containing a valid barcode file for all QC-passing cells, a feature file with ensembl gene ids and a matrix in the genes x cellsformat are generated. The filtered genes x cells matrix was further used as input for the data processing workflow.

Murine single-cell transcriptome data processing

The output from the Cell Ranger analysis framework was used as input to a custom analysis workflow, structured around the scanpy software toolkit in python (*70*) (https://scanpy.readthedocs.io/en/stable/). First, genes that were expressed (>=1 count) in <=3 cells across the whole dataset were removed (sc.pp.filter_genes with min_cells=3). Next, we filtered single-cells for i) counts (500 < total_counts < 35,000), ii) genes (1000 < n_genes < 6000) and iii) mitochondrial genes (pct_counts_mt < 10%). In addition, we used scrublet to remove potential doublets in our dataset (Data S1). To account for variable sequencing depth across cells, we normalized unique molecular identifier (UMI) counts by the total number of counts per cell, scaled to counts per 10,000 (CP10K; sc.pp.normalise_per_cell), and log-transformed the CP10K expression matrix (ln[CP10K+1]; sc.pp.log1p). Next and to generate cell type clusters, we selected the 2,000 most variable genes across samples by (1) calculating the most variable genes per sample and (2) selecting the 2,000 genes that occurred most often across samples (sc.pp.highly_variable_genes). After mean centering and scaling the

ln[CP10K+1] expression matrix to unit variance, principal component analysis (PCA; sc.tl.pca) was performed using the 2,000 most variable genes. To select the number of PCs for subsequent analyses, we used a scree plot and estimated the "knee/elbow" derived from the variance explained by each PC. Manual inspection of the UMAP embedding indicated sufficient intermixing of cell types across mice, highlighting no necessity for batch correction.

Differential gene expression analysis and cell cluster annotation for mouse tracheal samples

To determine the cellular identity of distinct clusters, we performed annotation based on the expression of known cell marker genes curated from the literature. Initial clustering was conducted using the Louvain algorithm with a resolution of 0.6 in the *FindClusters* function of the Seurat package (version 5.0.1). Differential gene expression for each cluster was assessed using *FindAllMarkers* function with the Wilcoxon rank-sum test and minimum log fold change of 0.25, comparing cells within each cluster against all other cells. These markers, alongside consensus marker genes reported across multiple publications, informed cell type annotation. Consensus marker genes included *Epcam*, *Krt5*, *Bcam*, *Wnt4*, *Trp63*, *Krt15*, *Epas1* (epithelial cells), *Itk*, *Skap1*, *Lck*, *Cd3e*, *Cd3d*, *Cxcr6*, *Cd3g*, *Cd3e*, *Icos*, *Il2ra* (T cells) and *Il1b*, *Alox5ap*, *Ctss*, *Mpeg1*, *Tyrobp*, *Mpeg1*, *Cd68*, *Cd74*, *Mef2c*, *H2-Aa* (macrophages). Marker gene expression was visualized using scanpy's "DotPlot" function. The high-level cell types assignments were further used for sub-clustering analysis.

Signature overlap of mouse epithelial cells

To assess the concordance between gene expression signatures from our mouse study (Data S3) and those reported in previous publications (Data S2) (*6, 7*), we conducted an overrepresentation analysis. This analysis was performed using a one-sided Fisher's exact test (fisher.test, alternative = "greater"). For each comparison, the top 50 genes from each reference dataset were used. If a dataset contained fewer than 50 genes, all available genes were included in the analysis. P-values from the Fisher's exact tests were adjusted for multiple comparisons using the Benjamini-Hochberg method (p.adjust). Unless otherwise specified, all functions were executed with default settings. This analysis provided a statistical basis to evaluate the overlap of gene sets, ensuring robust comparisons across studies.

Compositional analysis of murine airway with scCODA

To evaluate whether the abundance of any of the identified epithelial cell types changed by NTCU treatment, we used scCODA, a Bayesian model to assess compositional changes in pre-defined clusters from single-cell data (https://sccoda.readthedocs.io/en/latest/) (*24*). Using a hierarchical Dirichlet-Multinomal model, scCODA accounts for uncertainty in cell-type proportions as well as the negative correlative bias across cell-type proportions in relation to a reference cell type. Ciliated cells were used as reference for our analysis, however, it should be noted that the results did not change substantially when allowing scCODA to automatically determine the reference cell type. In addition to the reference cell type, we specified the treatment condition and the individual mouse as covariates. The remaining analysis was implemented as described in the single-cell best practice vignette (https://www.sc-best-practices.org/conditions/compositional.html).

Pseudotime trajectory analysis for murine samples

We employed Monocle2 (2.24.0) (*28*) for pseudotime analysis of basal, Krt4/Krt13^{+} and secretory cells. A single-cell trajectory was constructed using the Discriminative Dimensionality Reduction with Trees (DDRTree) algorithm, employing the top 400 significantly differentially expressed genes among the selected epithelial cell types. Cells were ordered along the trajectory with the state containing proliferative basal cells set as time zero, and pseudotime was calculated accordingly. To ensure clarity in trajectory dynamics visualization, cell numbers in each group were downsampled by 10%. Trajectory plots were generated using the *plot_cell_trajectory* function. The log2 fold change for cell abundance was computed for each cell type on each cell state, with sample size adjustments factored in using R.

Human trachea single-cell RNA-seq data pre-processing

10x raw data were processed with Cellranger v7.1.0 and aligned to the human genome reference GRCh38-2020-A. Expression matrices of each sample were cleaned from ambient RNA contamination using SoupX v.1.6.2 (*71*). During quality control, cells with fewer than 300 expressed genes were removed, as were those with log-transformed UMI counts per cell > 0.80. Cells expressing more than 10% mitochondrial genes and genes expressed in fewer than 0.1% of cells were also excluded. Doublets were identified and removed using DoubletFinder v2.0.4 (*72*). Additionally, immune cells were excluded from this study's analysis.

Signature overlap of human epithelial cell types

To evaluate the concordance between gene expression signatures from our human study and those reported in four previously published datasets, we performed an overrepresentation analysis. Specifically, we included proximal epithelial cell type-specific differentially expressed markers from Travaglini et al., 2020 (*33*), Goldfarbmuren et al., 2020 (*29*), and Deprez et al., 2020 (*10*). Additionally, markers enriched in hillock cells relative to basal and secretory cells were incorporated from Sikkema et al., 2023 (*32*) (Data S6). This analysis was conducted using a one-sided Fisher's exact test (fisher.test, alternative = "greater"). For each comparison, the top 50 genes from each reference dataset were utilized. If a dataset contained fewer than 50 genes, all available genes were included in the analysis. P-values from the Fisher's exact tests were adjusted for multiple comparisons using the Benjamini-Hochberg method (p.adjust). This analysis allowed us to quantify the overlap of gene sets, providing a robust and statistical framework for comparing gene expression signatures across multiple studies.

Human epithelial cell trajectory inference with Slingshot

We employed Slingshot 2.12.0 (*35*) to infer lineage trajectories within the basal, suprabasal and secretory cell compartments of the human airway surface epithelium. First, the Seurat object was converted to SingleCellExperiment format using *as.SingleCellExperiment*. To infer lineage trajectories, we ran *Slingshot* with start.clus = 'Basal cycling'. To evaluate shifts in cell fate and changes in progression speed, we used the *progressionTest* and *fateSelectionTest* functions from the condiments v1.4.0 package (*73*). Lineages were visualized on the batch-corrected UMAP, and differences in mean curve weights were plotted using R's ggplot2.

KRT4/KRT13 signature scoring

The top 50 differentially expressed genes identified in KRT4/KRT13 cells from our human single-cell epithelial data (Data S5) were used to compute signature scores. At the single-cell level, signature scores were calculated using the *AddModuleScore* function from the Seurat package (v5.0.1). These scores were visualized on the UMAP embedding using the *FeaturePlot* function from the same package. At the bulk-sample level, the signature score was computed using a previously published human dataset including samples normal airway epithelium, increasing grades of preinvasive disease and LUSC samples (*36*). Scores were derived from the average expression of normalized counts for the same top 50 differentially expressed genes. Visualization of the bulk-level scores was performed using R' ggplot2. Statistical significance of mean comparisons relative to the normal group was assessed using the Wilcoxon test.

Murine DNA sequence alignment

All DNA sequences were aligned to the GRCm38 reference genome by the Burrows–Wheeler algorithm (BWA-MEM) (*74*).

Removing germline variants (binomial filter)

To filter out remaining germline variants, we fitted a binomial distribution to the total variant counts and total depth at each SNV site across all samples from one patient. Thereby, the total depth at the position was used as the number of trials with the total number of variant counts as the number of successes. Germline and somatic variants were differentiated based on a one-sided exact binomial test, with the null hypothesis that the number of reads which support the variants across copy number normal samples is drawn from a binomial distribution where $p = 0.5$ ($p = 0.95$ for a copy number equal to one). In contrast, the alternative hypothesis posits that the reads are drawn from a distribution with $p < 0.5$ (or $p < 0.95$). Resulting p-values were corrected for multiple hypothesis testing using the Benjamini–Hochberg method and a cut-off was set at $q < 10^{-5}$ to minimize false positives. Variants for which the null hypothesis could be rejected were classified as somatic, while all others were classified as germline.

Removing errors (beta-binomial filter)

We filtered remaining artefacts by fitting a beta-binomial distribution to the variant counts and depths of all SNVs across samples from the same patient. In principle, the beta-binomial was used as it captures the difference between artefactual variant sites and true somatic variants. Thereby, artefacts often appear to be randomly distributed across samples and can be modelled as drawn from a binomial distribution. True somatic variants will be present at a high VAF in some samples, but absent in others, and are hence best captured by a highly overdispersed beta-binomial. For all variants, we quantified the overdispersion parameter (rho), with variants that had rho smaller than 0.1 being filtered out as previously described elsewhere (*2, 75*).

Clonality of samples

To estimate the clonal structure within a sample, we used a truncated binomial mixture model as described previously (*75*). The truncated distribution is used to reflect the minimum number of supporting reads ($n = 4$) that is required by CaVEMan. In theory, the model will try to separate the overall SNVs into the clones that they could have arisen from, each with their own

probability (VAF) and proportion (the amount of variants a clone contributes). The proportion of cells that inhabit a clone can be approximated by twice the estimated VAF of the clone.

Extraction of single-base pair substitution signatures

To identify mutational signatures for SNVs, we implemented the hierarchical Dirichlet process (https://github.com/nicolaroberts/hdp) on the 96 trinucleotide counts of all microdissected samples as well as to mutations assigned to each branch of the phylogenetic tree. The HDP was run with individual patients as the hierarchy, in 20 independent chains, for 40,000 iterations and with a burn-in of 20,000. The identified components were compared to existing signatures and components with ≥ 0.90 cosine similarity were considered identical. The remaining signatures were deconvoluted using an expectation-maximization algorithm, generally being explained by combinations of known signatures. In total, 9 signatures were evaluated which were then fitted back to the original mutation calls leveraging sigfit (https://github.com/kgori/sigfit).

Extraction of indel signatures

Indels detected in each sample were utilized to infer indel signatures using the MutationalPatterns package (*76*) in R. MutationalPatterns relies on non-negative matrix factorization to determine abundant signatures. All ID signatures detected previously (ID-1 - ID-18) (*77*), were utilized as input for signature discovery. In total, 15 signatures were identified across all samples, although some were only abundant due to low indel burden samples.

Analysis of driver variants

To systematically identify genes under positive selection in our dataset, we utilized dndscv in R (*78*). Initially, we assessed global dN/dS ratios of all genes which were found to be mutated in our dataset. Genes with q-value < 0.05 or p-value < 0.001 as well as highly recurrently mutated genes (≥ 8 unique mutations) were considered as driver genes. In addition, genes previously reported in lung cancer or normal bronchial epithelium were leveraged for subsequent analyses (n = 51 genes) (*2, 79-81*). For the tissue specific analysis, SNVs were split according to tissue of origin and leveraged as input for dndscv. Gene-level dN/dS ratios were used to assess tissue specific selection, reporting point estimates if the p-value of the mutation type of interest was smaller than 0.05.

Human WES alignment

Raw paired-end reads initial quality control (150 bp) was conducted using FastQC (v.0.11.9, https://www.bioinformatics.babraham.ac.uk/projects/fastqc/). This was followed by fastp (v0.23.2, flags: --qualified_quality_phred 28; --length_required 50 --length_limit 151) (*82*). Passed Raw quality control reads were aligned to the genome build (hg19), using Burrows-Wheeler Aligner (BWA-MEM) v0.7.17 (*74*). Unmapped reads and PCR duplicates were identified and marked using Picard tools v2.26.9 (http://broadinstitute.github.io/picard/). Aligned reads were Base quality score recalibrated (BQSR) using the GATK algorithm v4.2.0 (*61*). SAMtools v1.9 (*83*) and Maftools sampleSwaps (*84, 85*) were used to assess sample-sample correlation based on germline variations and to identify sample swaps and contamination events.

Extraction of mutational signatures in human WES

To estimate the contribution of various known mutational signatures in relation to clonality for each sample, we used the MutationalPatterns R package (*76*). The original mutation calls for each sample were annotated and divided into truncal or subclonal categories based on clonality inferred from phylogenetic analysis. We focused on assessing the contribution of the top six mutational signatures (SBS1, SBS2, SBS4, SBS5, SBS13, SBS92) identified in the genomic data of preinvasive lesions (*4*) and LUSC (*50*). For both truncal and subclonal mutations, the algorithm was run using a 6×96 matrix of these specific mutational signatures, ensuring that all mutations were attributed to one of these six signatures.

Lung cancer driver selection estimates in human WES

To evaluate positive selection for lung driver mutations in truncal and subclonal mutations derived from WES data, we employed the targeted dndscv model (*78*), focusing exclusively on genes covered by our WES target regions. We included only missense, nonsense, and splice-site mutations in our analysis. The assessment of positive selection was based on a curated list of potential lung cancer driver genes (n = 106) (Data S7) obtained from the Cancer Gene Census and previous publications (*4, 67, 80, 86*). Gene-level selection estimates, calculated using the dN/dS ratio, were used to evaluate selection pressure on truncal and subclonal mutations. Point estimates were reported if the global dN/dS > 1 and the global $q < 0.1$. A detailed list of the potential cancer driver mutations, sorted by clonality (truncal or subclonal), is provided in Data S9.

Supplementary Text

Here we provide details of the lattice-based models used to analyze the dynamics of basal cells in the trachea and airways under normal conditions and following NTCU treatment.

Model for the basal cell compartment

Clonal dynamics of basal cells in the trachea at homeostasis

Previous studies have shown that the trachea is maintained by a cellular hierarchy of basal cells that self-renew through a stochastic process of cell duplication and loss through differentiation (*8*). In homeostasis, stem cell loss and replacement must be balanced, so that the cell density remains approximately constant over time. In the basal layer, this results in a process of neutral cell competition, where all basal cells are equally likely to duplicate or become lost through differentiation.

To gain insight into how such neutral competition of basal cells finds a signature in their clonal dynamics, we first analyzed the mosaic labeling experiments of the trachea. Due to the high fraction of labeled cells (of the order of 50%), in the NTCU-treated trachea 24-weeks after tamoxifen administration, we observed the abundant merger of labeled and unlabeled clusters of cells. Thus, to quantify the dynamics of labeled cells, we focused on the organization of clusters of unlabeled cells or *voids*. By analyzing the cumulative distribution of sizes of voids for the control at 4 days and 24 weeks (168 days) after tamoxifen administration (Figure 2E,F), we noticed that at both time-points the cumulative distributions followed a power law-like decay with exponents close to -1. For the 4-day control, best fits to a power law dependence resulted in exponents of -0.8653 [-0.8647,-0.8658] 95% C.I. for the dorsal and -0.9112 [-0.9105,-0.9119] 95% C.I. for the ventral region. For the 24-week control, best fits to a power law dependence resulted in exponents of -0.7695 [-0.7687,-0.7702] 95% C.I. for the dorsal and -0.7966 [-0.7959,-0.7975] 95% C.I. for the ventral region.

Previous studies of neutral cell competition in the context of "voter" model dynamics have shown that the domain size distribution follows a power law decay with an exponent -2 (*21*), which translates to an exponent of -1 for the cumulative size distribution. Importantly, these results were obtained for a random and unbiased initial condition, with equal number of labeled and unlabeled cells.

Therefore, to model the clonal dynamics of basal cells in the trachea under normal conditions, we considered a process of neutral cell competition, representing the airway epithelium as a two-dimensional rectangular lattice of fixed size, reflecting the dorsal or ventral sides of the trachea, at homeostasis. Here, each lattice site corresponds to an individual basal cell that is either labeled or unlabeled, and that can duplicate stochastically at a given rate. The duplication process is coupled to the loss of one of its neighboring cells, thus maintaining a constant cell density over time. The cell being replaced is considered to have been lost through differentiation, thus exiting the basal cell compartment (see Figure 2D, top panel). Technically, this implementation of the model corresponds to a "reverse voter model" (or invasion process), and is a paradigmatic model for neutral competition dynamics (*87*) .

Clonal dynamics of basal cells in the trachea and airways following NTCU treatment

In NTCU treated samples, the distribution of void sizes was perturbed, the power law decay was lost, and the differences between the distribution of voids in the ventral and dorsal sides was enhanced compared to the control (Kolmogorov-Smirnov tests between dorsal and ventral

regions resulted in: p=0.058 for the 4-day control, p=3.45e-9 of the 24-week control and p=1.03e-15 for the 24-week NTCU-treated) (see Figure 2G). In the 24-week NTCU-treated samples we observed an increased proportion of large size patches of both labeled and unlabeled cells. These patches showed an enhanced cell density, as evidenced by the lack of empty spaces between cells (see, for example Fig. 2C) and a slight reduction in cell area (from 388 ± 83 pixels^2 [mean and SD] in the control to 370 ± 64 pixels^2 in the NTCU-treated samples, p=0.2326 from two-sided t-test, n=50 cells per condition).

In the context of the neutral model, there is only one dimensionless control parameter for the dynamics corresponding to the product of the basal cell division rate, r_{basal}, and time post-induction, t, which provides an estimate for the average number of divisions each cell has gone through. As we consider a homogeneous population of basal cells, a global change in the average cell division rate would not lead to a change in the power law-like dependence or exponent of the void size distribution, as this would amount to a simple rescaling of time (*21*). As such, the neutral model alone could not provide an explanation to the shift in the void size distribution resulting from long-term exposure to NTCU. This suggested that the dynamics in the NTCU-treated samples was not driven by a homogeneous population of proliferative cells. Based on this, and the observed increase in cell density of the basal layer of the NTCU treated samples, we hypothesized that a subpopulation of basal cells loses their ability to differentiate and exit the basal compartment, thus overcrowding the basal layer, and outcompeting the surrounding normal-like cells. Such differentiation impairment is supported by the measured reduction in secretory cell coverage from our SCGB1A1 marker data (see Figure 3H, J). We refer to this subpopulation as "fitter mutant" cells.

As a minimal model for this dynamic, we considered that, among the broad population basal cells, a small fraction became fitter after exposure to NTCU. These fitter cells compete non-neutrally with their neighboring normal-like basal cells meaning that, whenever fitter mutant cells divide, they preferentially replace a neighboring normal-like cell, whereas normal-like cells are unable to replace fitter mutant ones. Fitter mutant cells, on the other hand, compete neutrally with each other. This dynamic implies that fitter mutant clones expand and colonize tissue through the loss of neighboring normal-like cells.

In the following, we describe the numerical implementation of both the neutral and non-neutral models, and discuss the comparisons of the model with the experimental data.

Numerical implementation

Neutral competition model

To simulate the neutral competition model for the dynamics of normal trachea, we considered a two-dimensional rectangular lattice of fixed size with $N = 300 \times 100$ sites, where each site corresponded to a single basal cell (see Figure S3B). As we processed dorsal and ventral regions separately, we considered closed boundary conditions in our simulations. Considering that all N cells in the system duplicate symmetrically with an equal rate r_{basal}, the stochastic loss-replacement dynamic follows a standard Gillespie dynamic (*88*):

1. A single cell (regardless as to whether it is labeled or unlabeled) is chosen at random.
2. The chosen cell duplicates, replacing one of its four nearest neighbor cells, also selected at random.
3. The time t is updated to $t + \tau$, with $\tau = -\frac{1}{\omega} log(q)$.

Here, $q \in (0,1]$ is a uniformly distributed random number, and $\omega = Nr_{basal}$ is the propensity function. In all simulations, the division rate of cells was fixed to an arbitrary value of 0.1 $[\text{time}]^{-1}$, such that 10 units of time corresponds to the typical time between two consecutive duplication events. Simulations were run until a sufficiently long run time $t = T_{max}$ was reached.

Our preliminary numerical studies of the model showed that the initial fraction and spatial distribution of labeled cells have an effect on the details of the resulting distribution of void sizes. To account for these variations in our simulations, we considered as initial labeling conditions the configuration of labeled cells observed in the 4-day labeling control experiments. For this, we considered a central region of the dorsal and ventral sides for 2 of the control samples that covered approximately 40% of the whole trachea, which amounted to a domain of 300×100 sites. For constructing the initial conditions, we considered the fully resolved images of the dorsal and ventral regions of the trachea, after rescaling by the typical cell size and thresholding, we obtained a binarized 300×100 pixel image, where each pixel (or site) represents an individual basal cell (see Figure S3B), this was validated by visual inspection. We ran 200 realizations of the model in total, 50 for each of 2 dorsal and 2 ventral initial conditions, which were then averaged to compute the mean and standard deviation shown in Figure 2F. We note here that increasing the system size did not significantly change the results of the model.

NTCU-treatment model

As before, to simulate the two-dimensional non-neutral model for NTCU-affected basal cells in the trachea, we considered a lattice of fixed size $N = 300 \times 100$ sites, where each site corresponded to a single basal cell. As before, boundaries were closed and initial conditions constructed from the labeling control data (Figure S3B). However, in this case, we considered that initially, only a fraction f_p of all the cells were fitter mutants, while the rest of the cells where normal-like mutant cells that had no competitive advantage over fitter mutant ones. Fitter mutant cells were chosen at random and could be either labeled or unlabeled. As before, considering an initial number Nf_p of proliferative cells that propagate on a background of non-dividing cells, the cell dynamic follows from the following set of rules:

1. A fitter mutant cell is chosen at random.
2. The chosen cell divides symmetrically:
    - 2.1. If at least one of its 4 nearest neighbors is a normal-like cell, then one of them is chosen at random and replaced by the daughter of the fitter mutant cells.
    - 2.2. If the chosen cell is surrounded by fitter mutant cells, then a neighbor is picked at random and replaced.
    - 2.3. The number of proliferative cells is updated accordingly.
3. The time is updated as before.

Here, we considered the limiting case in which the expansion of fitter mutant cells is much faster than that of normal-like cells, so that there is no need to account for the normal cell dynamics. However, the results remain unchanged if normal-like cells are allowed to turnover,

as long as they do not compete weakly with fitter mutant cells, in which case they would only slow down the invasion process. In the long term, normal-like cells make no difference for the distribution of void sizes, as all normal cells are ultimately removed by mutant ones. Additionally, we note here that increasing the system size does not change the resulting distribution of voids, as long as the fraction of mutant cells f_p is kept constant. Thus, this model has two free parameters: the dimensionless parameter $r_{basal}t$, and f_p. As mutant clones proliferate freely until all normal-like cells are expelled, the average clone size when the system is fully covered by mutant cells is approximately $1/f_p$, ignoring clone loss due to neutral competition between mutant clones. In our mosaic labeling, we do not have access to clonal information. However, we considered the average void size in the trachea of 141 ± 54 cells as a guide for our estimate of f_p.

When applying the model to the airways, we considered a domain of dimension $N = 350 \times 100$ sites, which corresponded to a whole bronchus, as estimated from the typical length and diameter of a bronchus and typical size of a basal cell. Here, periodic boundary conditions along the short axis were considered to account for the circular shape of the bronchus. In this case, the initial conditions were constructed to emulate invading clones from the trachea. For this, we seeded clones along the proximal (long axis) of the domain (see Figure S12A, left panel), where each clone was assigned a different starting size in order to account for slightly different timings of invasion into the airway.

Comparison of the model with experiment

Control conditions

Given the measured initiation conditions, the neutral competition model captured accurately the void size distribution at 24-weeks labeling controls. We found that there was a range of parameters for which the model provided a good fit to the data, e.g., for $r_{basal}t$ in the range $[1, 3.0]$ the cumulative distributions varied slightly, with R^2 varying between [0.92, 0.96] for dorsal and remaining around 0.95 for ventral (see Figure 2F and Figure S3C). In Figure 2F the curve for $r_{basal}t = 2.5$ is shown (R^2=0.96 for both ventral and dorsal). These results indicate that an average of one round of symmetric basal cell division is already sufficient to account for the shift in the void size distribution from an initial power law-like decay with an exponent close to -1 at 4-days post-labeling to values above -0.8 at 24-weeks post-labeling. Note that these findings emphasize the importance of choosing appropriate initial labeling conditions, with variations in the labeling efficiency altering the evolution of the void distribution. These results for the expansion capacity of basal cells are remarkably consistent with the slow turnover of basal cells reported in previous work (*8*). There, the loss/replacement rate was reported as once per 26 weeks, with the vast majority of basal cell divisions resulting in asymmetric fate outcome, leaving the basal clone size unchanged. Here, for simplicity, we have ignored the cellular hierarchy of the differentiating secretory cell types produced by the renewing basal cell population.

NTCU treatment

The non-neutral model was then applied to model the dynamic of mutant basal cells in the trachea and airways. First, considering a fraction $f_p = 0.01$, resulting in around 1/300 initial fitter mutant cells in the trachea, the model provided a good fit to the void size distribution of the 24-week NTCU-treated samples when considering $r_{basal}t = 13$, both the dorsal (R^2 =0.91) and ventral (R^2 =0.90) regions. This suggests that mutant cells expand in the basal layer

approximately 13 times faster than predicted by the loss/replacement rate of normal cells by the neutral model. Considering the 24-week time span (or 168 days), this value suggests a duplication rate of once every 13 days, which is comparable to the estimated cell division rate of basal cells in the normal mouse trachea (*8*). These results were also consistent with our EdU incorporation (see Figure S3E) and SCGB1A1 marker data (see Figure 3H,J), which showed no significant change in proliferation compared to control, and a reduction in differentiated secretory cells. In addition to the quantitative predictions, the non-neutral dynamics reproduced some of the qualitative features of the mosaic labeling, including the existence of large irregular voids, which in the model originate from the merger of multiple neighboring clones (see Figures S3A and S3D).

When studying the airways, we lacked quantitative information regarding the void size distribution. In the airways, the invading front, i.e. the interface between the expanding mutant population that invaded the airways and the normal background, showed a rough boundary, characteristic of a stochastic growth process (see Figure S2). Furthermore, clonal labeling of cells in the airways showed that submerged mutant clones, defined as clusters of cells labeled in the same color surrounded by unlabeled mutant cells, could continue expanding. These clones were observed to fragment into multiple clusters of cells (see Figure S12B). To assess whether the non-neutral dynamic could capture some of these morphological traits, we simulated the invasion process in a bronchus-shaped domain, allowing fitter mutant to invade the airway tissue from the trachea (see Figure S12A). As labeled mutant clones propagated through the bronchus, we first noted that the leading edge became progressively rough over time, as expected from a stochastic growth process. As clones propagate through the airway, the competition between neighboring mutant clones causes the detachment of some clones from the leading edge (see Figure S12C), as seen in the confetti labeling experiments (see Figure S12B). Moreover, the boundary between mutant clones showed signs of fragmentation, with small groups of cells becoming disconnected from their clones (see Figure S12C). This shows that the bulk of mutant clones is dynamic and driven by competition between neighboring mutant cells.

Moreover, from the whole-genome sequencing analysis (see discussion related to Figure 6), we noted that the clonal composition in distal airways tended to have a more homogeneous genetic signature than proximal airways, suggesting a loss of clonal diversity as clones move from proximal to distal regions of the tissue. To explore the origin of this behavior we analyzed the results of our simulations at the time when clones had invaded the whole bronchus (see Figure 12A, right panel). Specifically, we measured the number of distinct clones (averaged over 32 realizations) as a function of the distance from the proximal boundary (see Figure S12D). We observed that, regardless of the initial number of invading clones, the number of distinct clones is reduced dramatically, from 16-80 initial clones down to around 10 distinct clones as they propagate through the airway. Although the invasion process originates from the non-neutral competition between mutant clones and normal-like cells, the reduction in clone number occurs due to the neutral competition between neighboring mutant clones. This competitive dynamic continues once the airway is fully spanned by mutant clones, so that the number of clones can be further reduced over time and along the length of the airways.

Testing the effect of driver mutations

The reduction in clonality in the airways can be accelerated by considering the presence of driver mutations in a fraction of the invading mutant clones, as suggested by our sequencing data (see discussion around Figure 5). Here we refer to these clones as *driver clones*. For simplicity, in our model we considered the case in which only one of all mutant clones had an

expanding advantage over neighboring mutant clones (see white clone in Figure S12E). Consistent with our scRNA-seq analysis (see discussion around Figure 3), this advantage was not incorporated as an increase in proliferation of the driver mutant clones, but as an increased probability of remaining in the basal layer. This rule allows the driver clone to outcompete neighboring mutant cells, until covering the whole airway. We note that in the non-neutral model without clonal drivers, the time of consensus, i.e. the time it takes for the airway to become covered by a single clone is expected to grow $Nlog(N)$ (*89*), with N being the system size. On the other hand, in the model with driver mutations, the time of consensus grows linearly with the system size N and is proportional to the advantage of driver clones over mutant clones. Thus, driver mutations could allow a faster convergence towards monoclonality in the airways and the trachea.

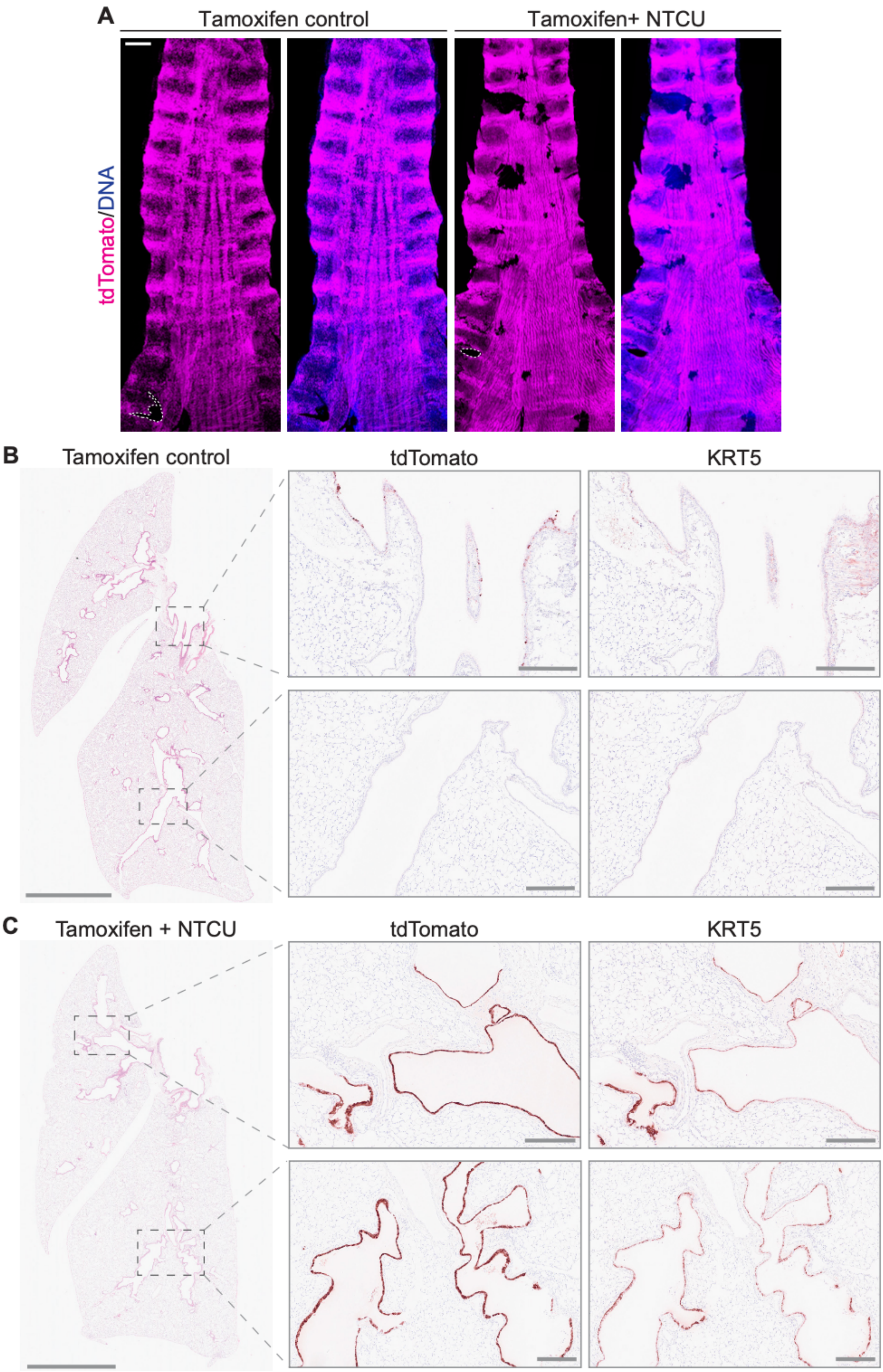
A
Tamoxifen control
Tamoxifen+ NTCU
tdTomato/DNA
B
Tamoxifen control
tdTomato
KRT5
C
Tamoxifen + NTCU
tdTomato
KRT5

Fig. S1. NTCU-induced preinvasive disease originates from basal cells.

(A) 3D projections of dorsal trachea whole-mounts from control and NTCU-treated *KRT5-CreER*;*tdTomato* mice 24 weeks after high-density basal cell labeling. The dorsal smooth muscle runs longitudinally between the open cartilage rings, whose dorsal ends can be seen at the lateral edges of the preparation. Scale bar, 500 µm.

(B) Hematoxylin and eosin (H&E) staining (left) and immunohistochemistry (IHC) for tdTomato and the basal/squamous cell marker KRT5 on sequential lung sections from a *KRT5-CreER*;*tdTomato* mouse treated only with tamoxifen. Cells expressing tdTomato and KRT5 are restricted to the most proximal part of the bronchial epithelium (top panel). Scale bars, 2.5 mm (H&E), and 250 µm (IHC).

(C) H&E staining (left) and IHC for tdTomato and KRT5 on consecutive lung sections from a *KRT5-CreER*;*tdTomato* mouse sequentially treated with tamoxifen and NTCU. Lineage-labeled tdTomato^{+} cells can be observed along the bronchial tree. The expression domain of KRT5 matches that of tdTomato. Scale bars, 2.5 mm (H&E), and 250 µm (IHC).

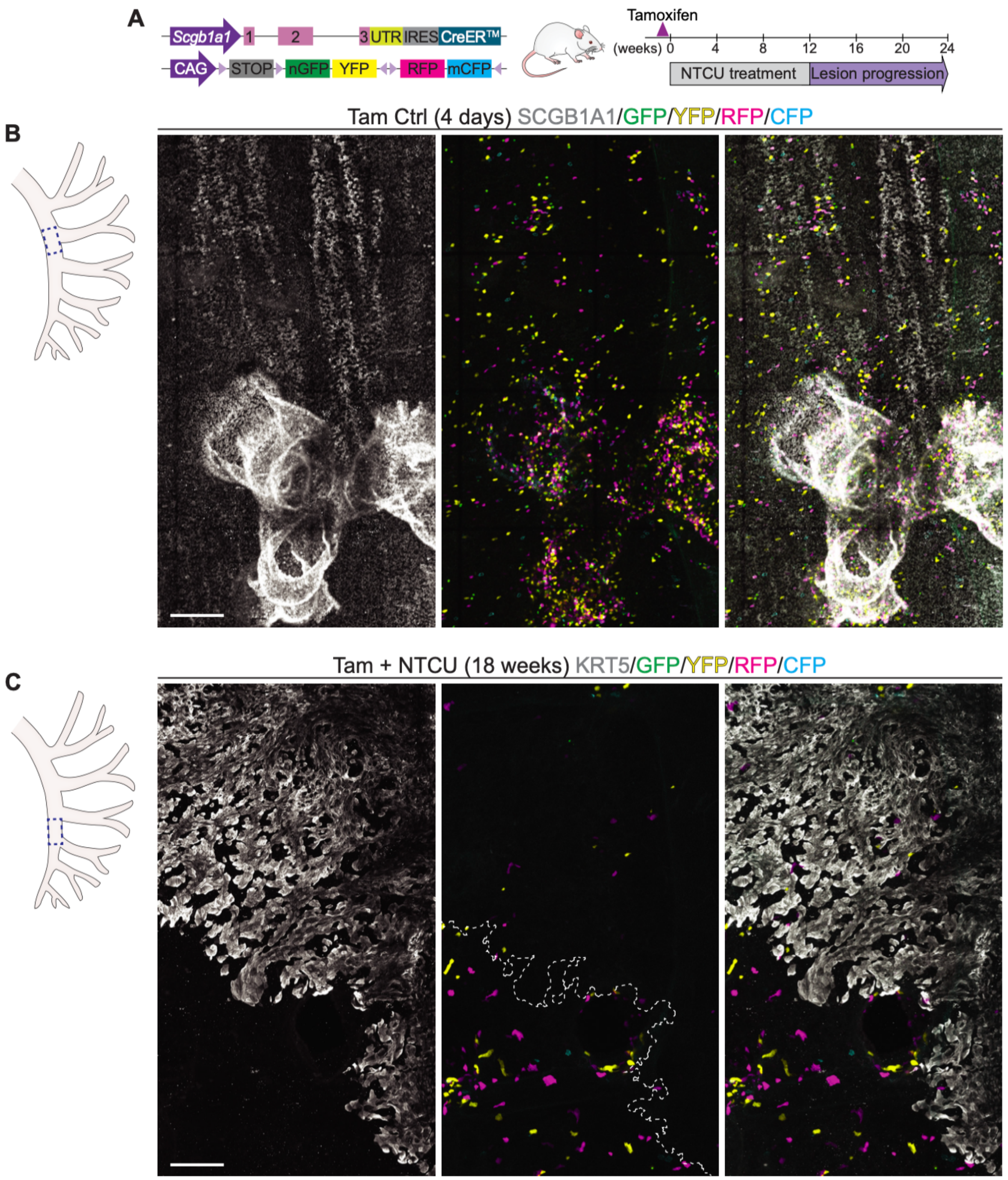

Fig. S2. *Scgb1a1*-expressing secretory cells do not contribute to NTCU-induced carcinogenesis.

(A) Strategy to track SCGB1A1^{+} secretory cells during NTCU-induced carcinogenesis in *Scgb1a1-CreER*^{TM};*R26R-Confetti* mice. Mice received a daily dose of tamoxifen for 4 days to label *Scgb1a1*-expressing cells prior to NTCU treatment.

(B) 3D projection of lung whole-mount showing that bronchial Confetti^{+} lineage-labeled cells express the secretory cell marker SCGB1A1, 4 days after tamoxifen administration. The schematic on the left indicates the anatomical location of the region shown to the right. Scale bar, 200 µm.

(C) 3D projection of lung whole-mount from a *Scgb1a1-CreER*^{TM};*R26R-Confetti* mouse sequentially treated with tamoxifen and NTCU, 18 weeks after tamoxifen administration. Clones derived from Confetti^{+} *Scgb1a1*-lineage-labeled cells do not express the

basal/preinvasive squamous cell marker $KRT5^+$ and are enriched in bronchial areas showing no signs of NTCU-induced disease. The schematic on the left indicates the anatomical location of the region shown to the right. Scale bar, 200 μm.

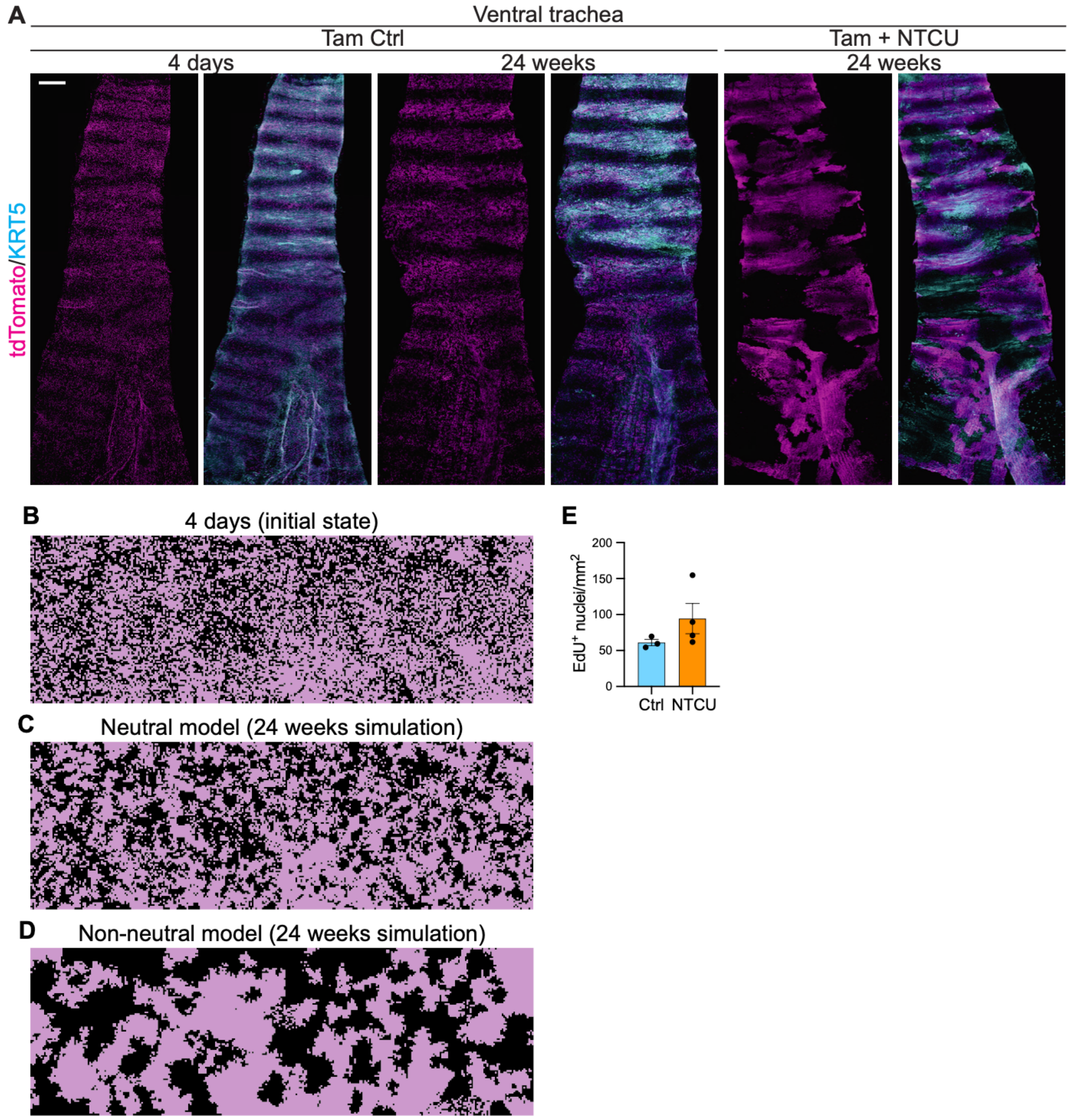

Fig. S3. NTCU treatment induces basal cell clonal expansions.

(A) 3D projections of ventral trachea whole-mounts from control and NTCU-treated *KRT5-CreER*;*tdTomato* mice at 4 days and 24 weeks post-tamoxifen. Scale bar, 500 µm.

(B) Representative image of the initial condition used in the numerical simulations of the neutral and non-neutral models, obtained from images of the trachea 4 days post-tamoxifen. Pink and black regions correspond to labeled and unlabeled cells, respectively.

(C) Representative image of a simulation of the neutral model in the trachea (system size 300 × 100 cells) after approximately 1 turnover of the basal cell compartment (see Supplementary Text for details).

(D) Representative image of a simulation of the non-neutral model after approximately 13 turnovers of the basal cell compartment (see Supplementary text for details).

(E) Number of EdU^+ cells per mm^2 of tracheal epithelium in control and NTCU-treated mice, following a 24h chase. Analyses were done 24 weeks after NTCU commencement on dorsal trachea whole-mounts. Bars depict mean ± SEM. Dots represent values from individual mice. Unpaired two-tailed *t*-test with Welch's correction indicated no statistically significant differences between groups.

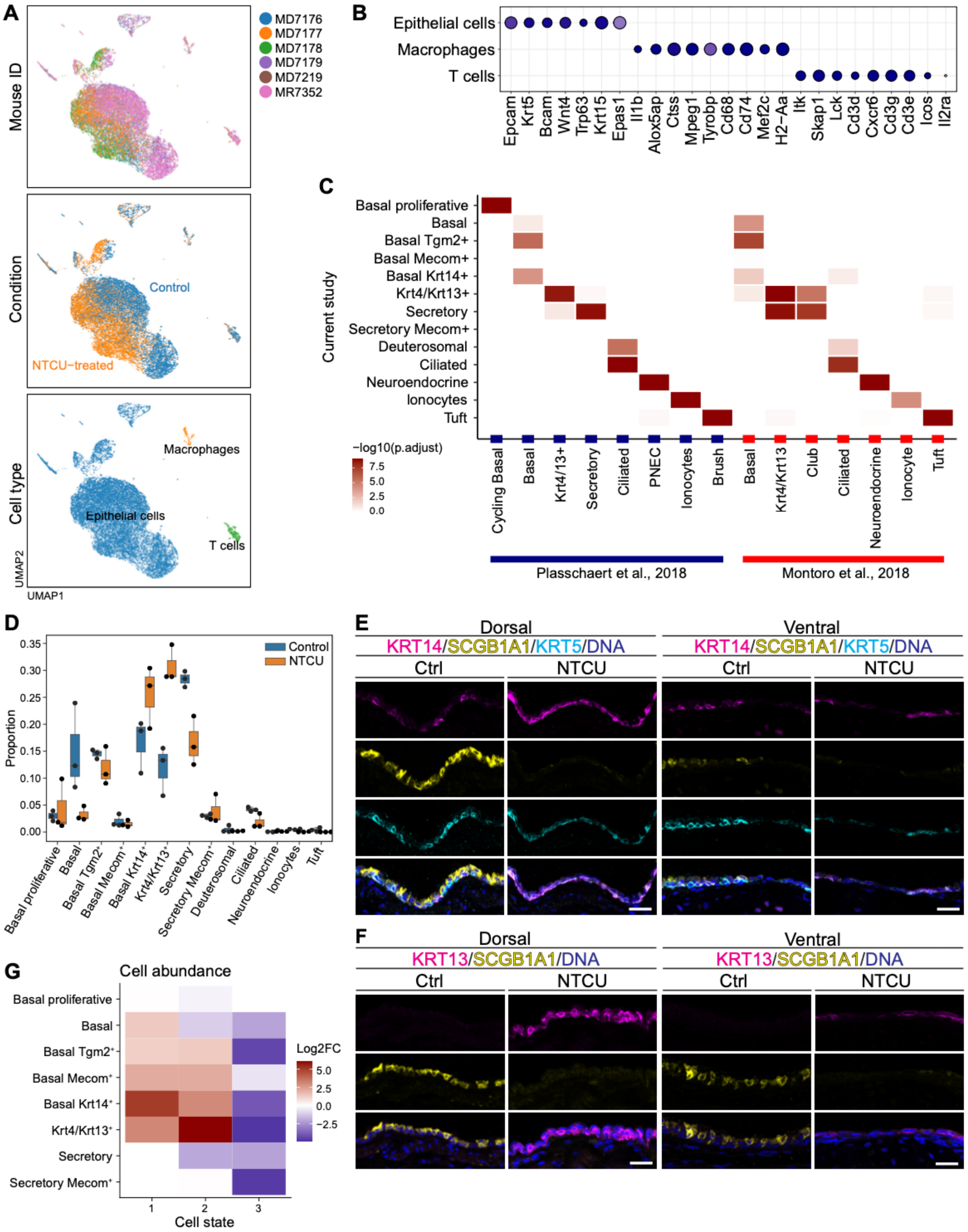

Fig. S4. Cell type signature scoring and NTCU-induced cell changes.

(A) UMAP visualizations colored according to mouse id (top), condition (middle) and cell type (bottom) for all tracheal cells (30,020) isolated from NTCU-treated and control mice, 15 weeks after treatment commencement.

(B) Dotplot depicting the expression of selected marker genes for cell types shown in A.

(C) Cell type signature overlap analysis comparing the different murine airway epithelial cell types/states identified in the current study with those described in previous scRNA-seq analyses (*6, 7*).

(D) Boxplot highlighting the abundance of each epithelial cell type in NTCU-treated (15 weeks) and age matched controls. Individual donors are represented by black dots.
(E) Immunofluorescence for the basal markers KRT14 and KRT5 and the secretory cell marker SCGB1A1 on trachea sections from control and NTCU-treated mice, 15 weeks after treatment commencement. Scale bar, 25 μm.
(F) Immunofluorescence staining for KRT13 and SCGB1A1 on trachea sections from control and NTCU-treated mice, 15 weeks after treatment commencement. Scale bar, 25 μm.
(G) Heatmap displaying log2 fold changes in the relative abundance of cell types between NTCU-treated and control groups across the three cell states identified by the trajectory analysis.

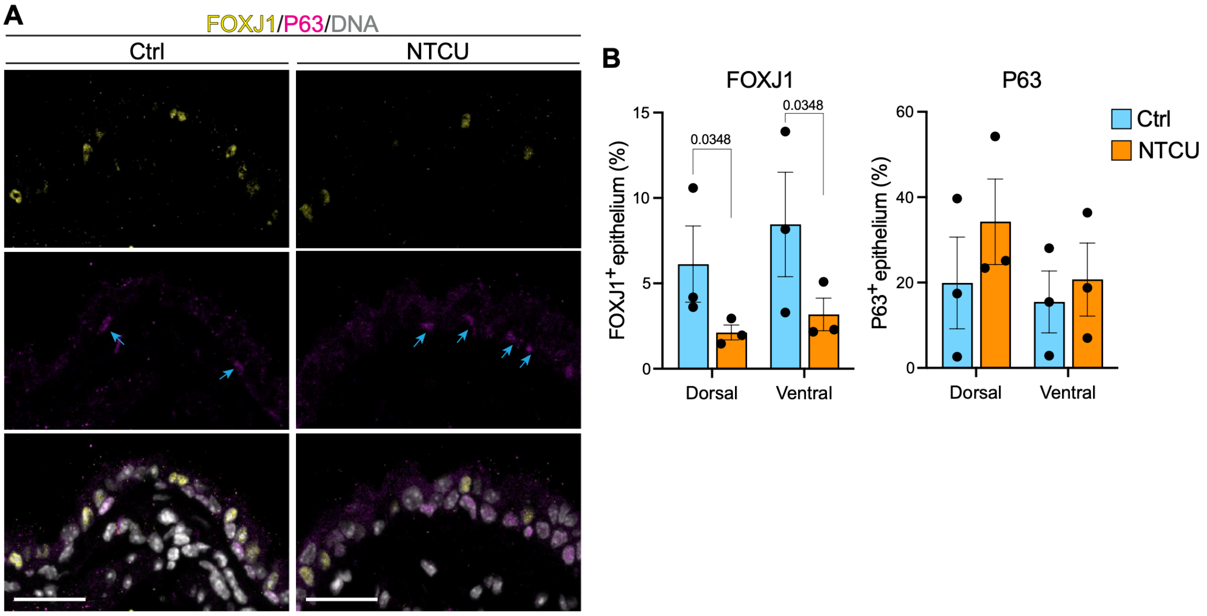

Fig. S5. Effects of NTCU on tracheal epithelial cells.

(A) Immunofluorescence staining for the ciliated cell marker FOXJ1 and basal cell marker P63 on tracheal sections from control and NTCU-exposed mice, 15 weeks after NTCU treatment commencement. Arrows point to $P63^{+}$ nuclei. The dorsal epithelium is shown. Scale bars, 25 µm.

(B) Quantitative analyses of FOXJ1 and P63 expression in the dorsal and ventral tracheal epithelium, 15 weeks after the start of the experiment. Bars depict mean ± SEM. Values from individual mice are shown. Statistically significant differences are indicated by the *p* values (two-way ANOVA).

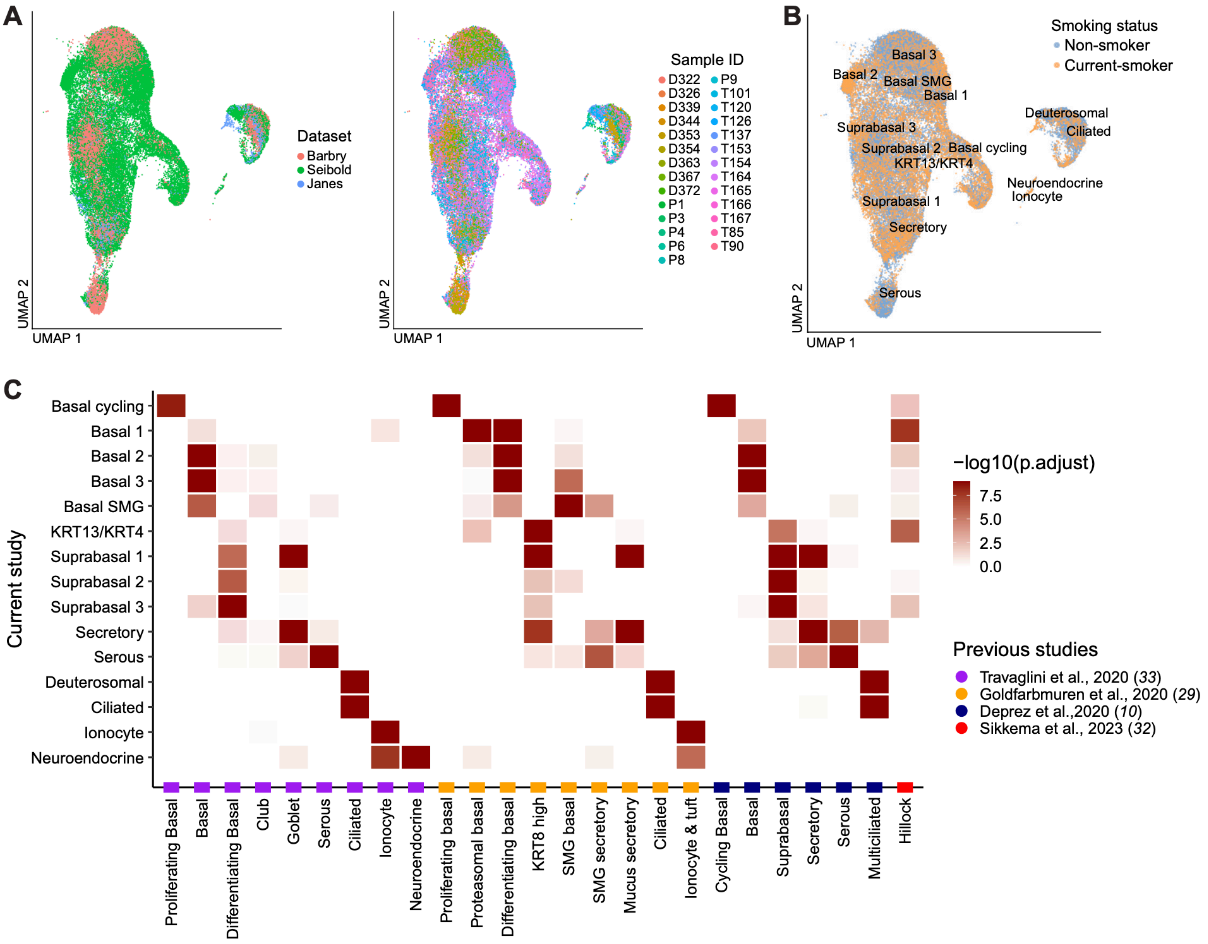

Fig. S6. Single cell profiling of human tracheal epithelium.

(A) UMAP visualizations showing the three datasets (left) and all trachea samples from a total of 18 non-smokers and 9 current-smokers (right) included in the study.

(B) UMAP visualization depicting cells by donor's smoking status.

(C) Cell type signature overlap analysis comparing the different human tracheal airway epithelial cell types/states identified in the current study with those described in previous scRNA-seq analyses of the human airways (*10, 29, 32, 33*).

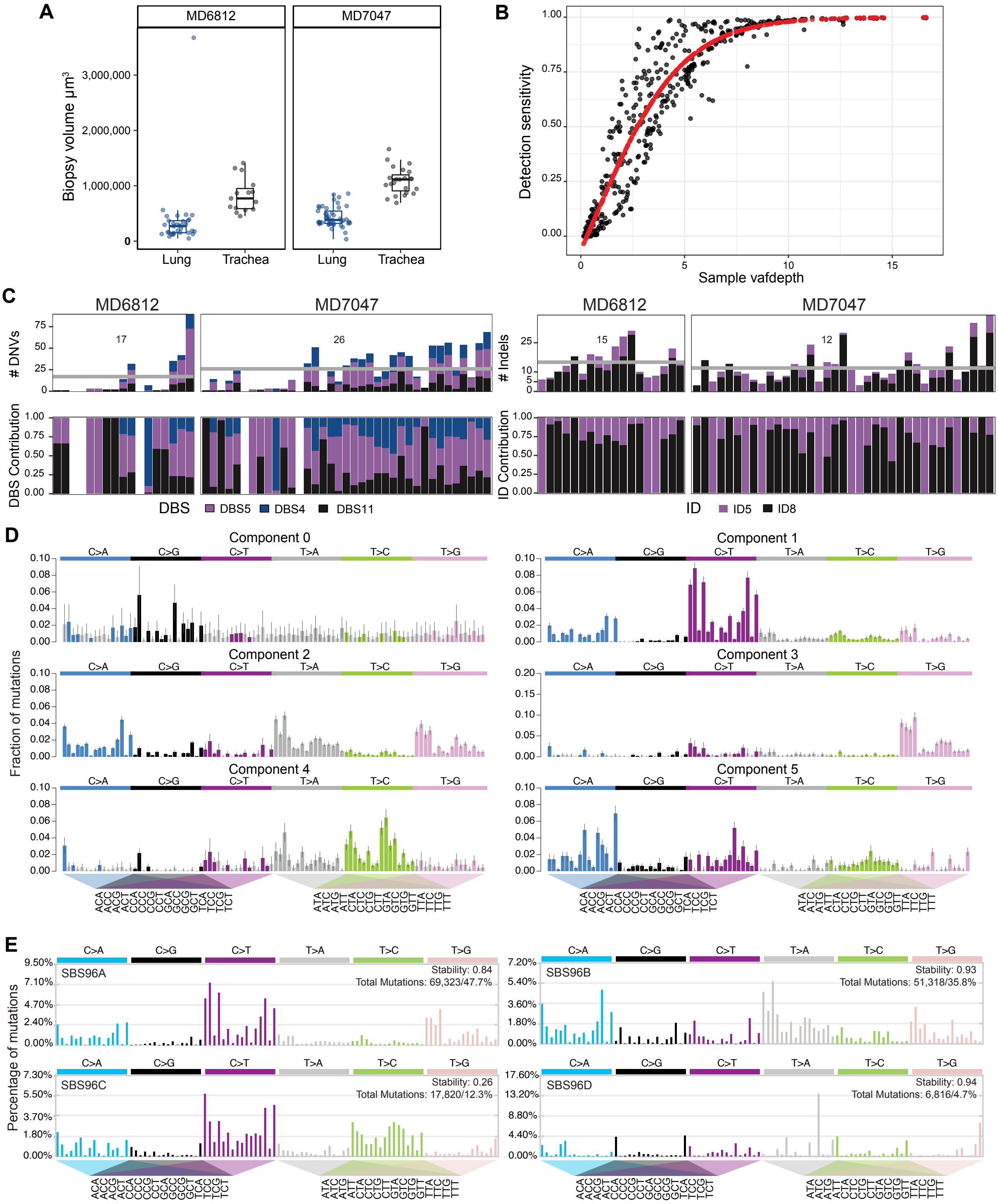

Fig. S7. Genomic alterations and signatures in the murine airway epithelium.

(A) Volume of dissected microbiopsies across both mice, split according to anatomical location.

(B) Dotplot showing the relationship between the detection sensitivity for SNVs (y-axis) as a function of the variant allele fraction per clone and coverage of the sequencing per sample (x-axis). The red line denotes the sensitivity fit for the correction of detected mutations.

(C) Burden of dinucleotide nucleotide variants (DNVs) and insertion and deletions (indels) with the respective fitted mutational processes (DBSs, IDs), across clones detected in both NTCU-treated mice. The order is equivalent to Figure 5A. Stacked bar plots showing the proportional contribution of each mutational signature to the respective genomic alteration. The grey line highlights the average burden across clones per mouse.

(D) Extracted components from mutational signature analysis using HDP.
(E) Extracted components from mutational signature analysis using sigProfiler.

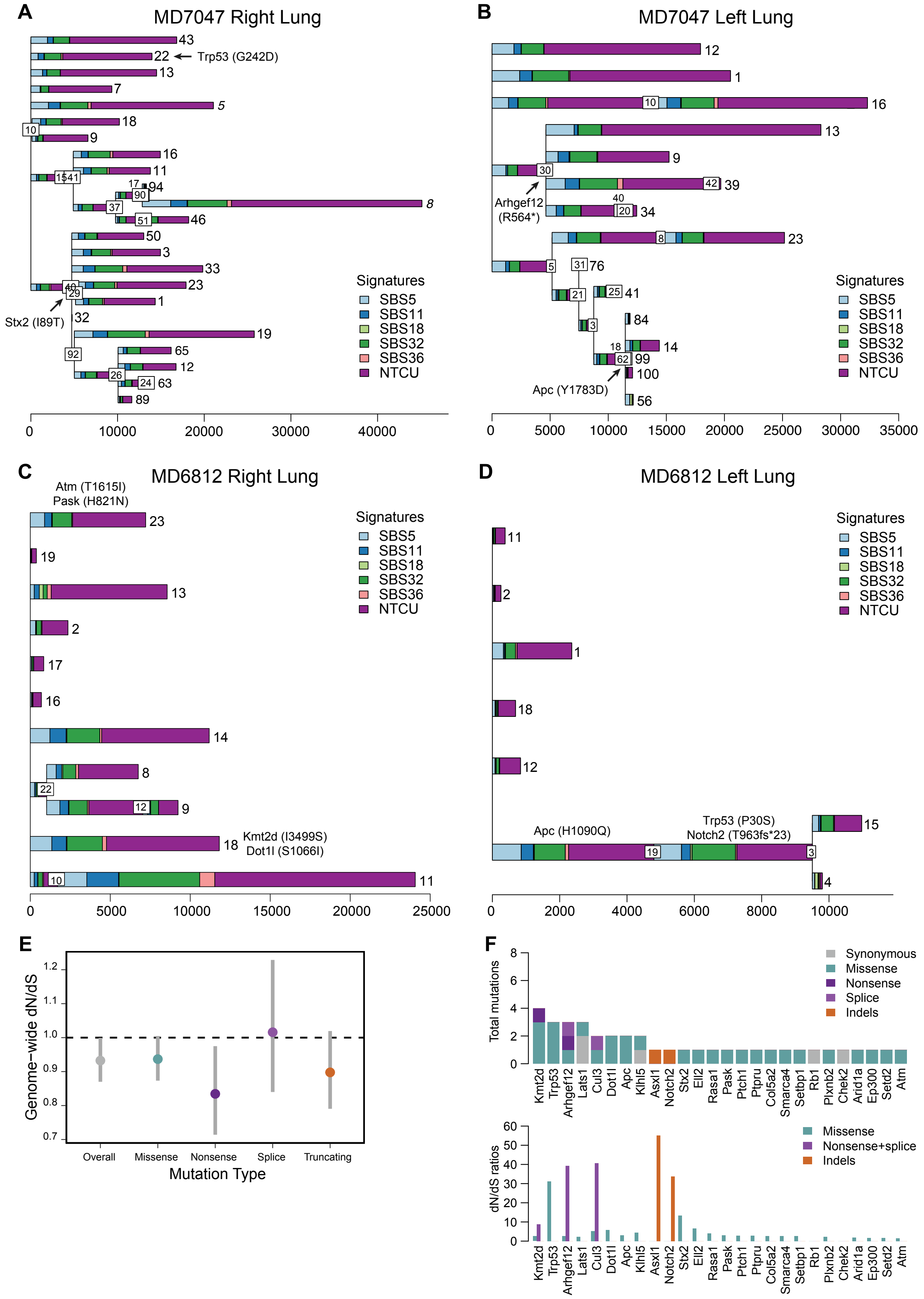

Fig. S8. Analysis of murine airway epithelial clones in two NTCU-treated individuals. **(A)** Phylogenetic tree for all samples and clones located on the right lung of mouse MD7047. Clones are highlighted with individual numbers, with mutations colored according to the mutational signature contributing to each branch. The boxed numbers represent progenitors of

the clones branching of the respective box. Where boxes overlap, the clone number is displayed above the box. Selected mutations in driver genes are annotated on some branches including the amino acid change.
(B) Phylogenetic tree for all samples and clones located on the left lung of MD7047
(C) Phylogenetic tree for all samples and clones located on the right lung of MD6812.
(D) Phylogenetic tree for all samples and clones located on the left lung of MD6812.
(E) Genome-wide dN/dS ratios for all mutations and divided by the respective impact.
(F) Top, barplot showing the number of unique mutations for mouse homologs of known squamous cell carcinoma driver genes (see Methods). Bottom, barplot depicting selection coefficients (dN/dS ratios) for mutation type categories per gene.

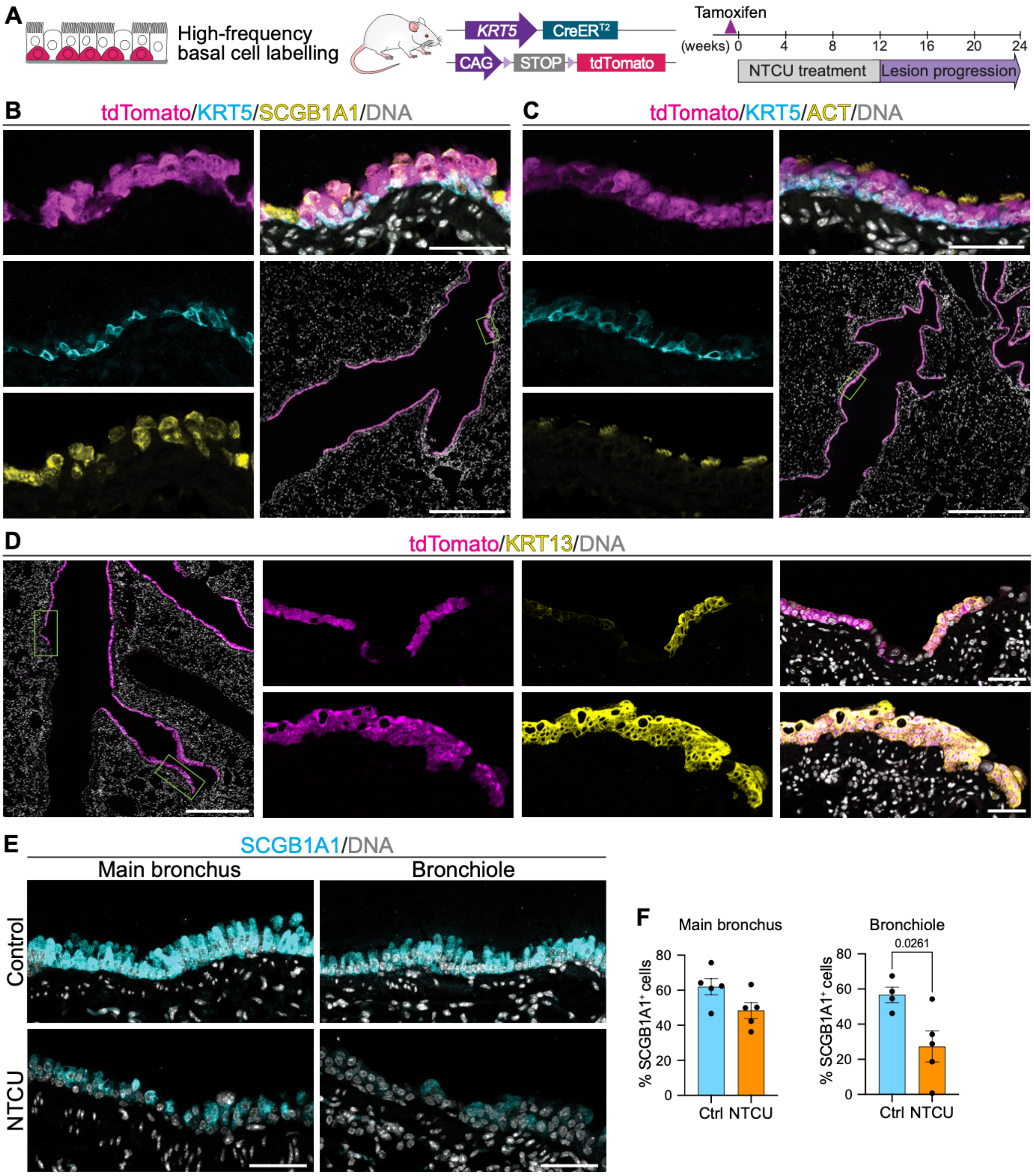

Fig. S9. Basal cells colonize the intrapulmonary airways following NTCU treatment.

(A) Strategy to track lineage-labeled airway basal cells in the intrapulmonary airways following NTCU treatment of *KRT5-CreER*;*tdTomato* mice.

(B) Immunofluorescence images of lung tissue sections from *KRT5-CreER*;*tdTomato* mice sequentially treated with tamoxifen and NTCU. Expression of the basal cell marker KRT5 and the secretory cell marker SCGB1A1 is seen in subpopulations of tdTomato^{+} lineage labeled cells in the intrapulmonary airways. Scale bars, 50 µm (high magnification images); 500 µm (tissue overview).

(C) Immunofluorescence images of lung tissue sections from *KRT5-CreER*;*tdTomato* mice sequentially treated with tamoxifen and NTCU. Subpopulations of tdTomato^{+} lineage-labeled cells in the intrapulmonary airway express basal (KRT5) or ciliated (ACT) cell markers. Scale bars, 50 µm (high magnification images); 500 µm (tissue overview).

(D) Immunostaining for tdTomato and KRT13 on lung sections from *KRT5-CreER*;*tdTomato* mice sequentially treated with tamoxifen and NTCU. The left panel displays a tissue overview indicating the location of the regions presented to the right. Scale bar, 500 µm. The top panel shows the advancing front of the tdTomato^{+} population in the main bronchus. The bottom panel displays a squamous lesion in one of the bronchioles. Scale bars, 50 µm (high magnification images).
(E) Antibody staining for the secretory cell marker SCGB1A1 on lung tissue sections from control and NTCU-treated mice, 24 weeks after the start of the experiment. Representative images of the epithelium lining the intraparenchymal main bronchus and a bronchiole are shown. Scale bars, 50 µm.
(F) Quantification of the proportion of total epithelial cells expressing SCGB1A1 in the first half of the intraparenchymal main bronchus (left) and the fourth bronchiole of the left lung (right), 24 weeks after the start of NTCU treatment. Bars depict mean ± SEM. Each dot represents a different individual. Statistically significant differences are indicated by the *p* value (unpaired two-tailed *t*-test with Welch's correction).

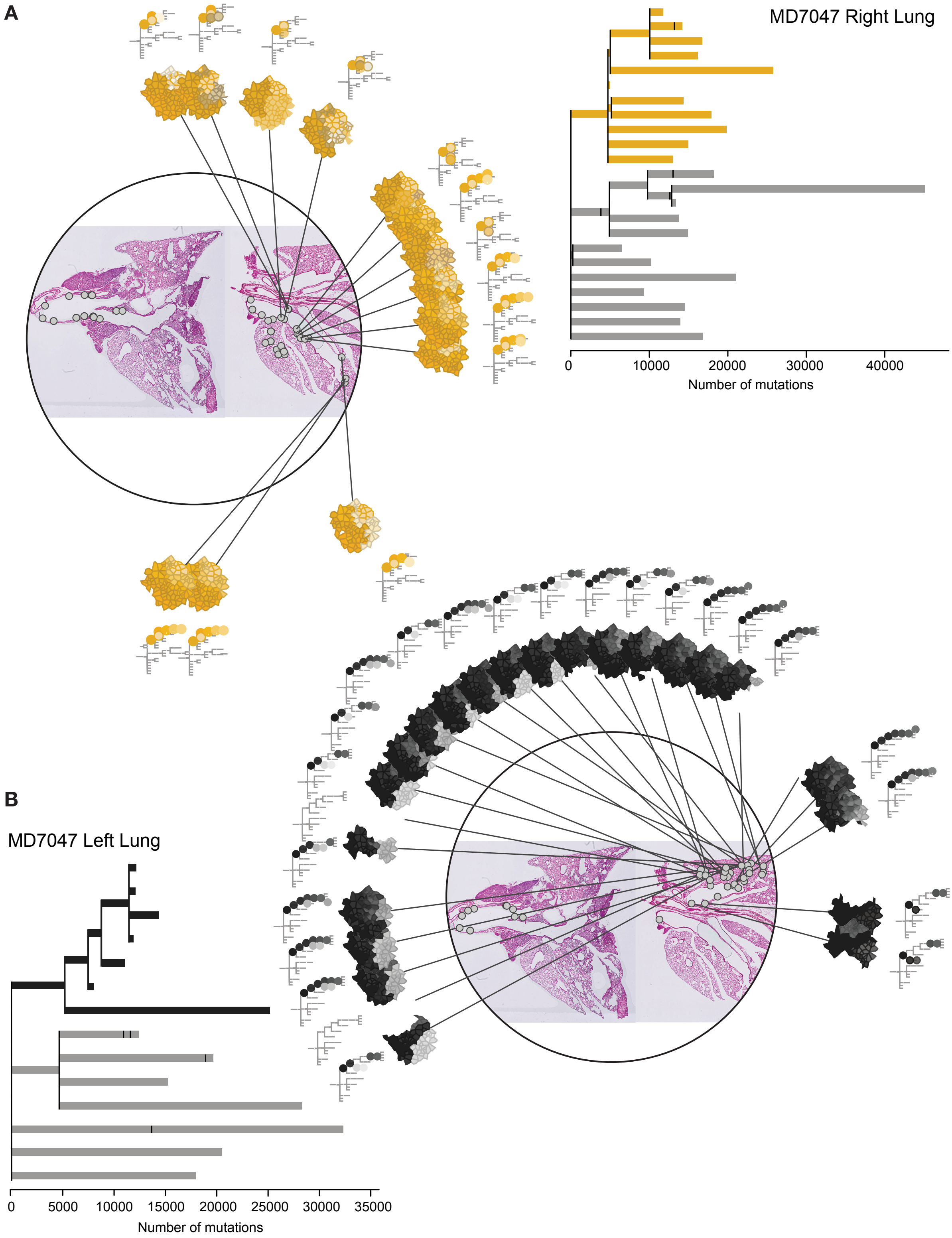

Fig. S10. NTCU-driven clonal expansions in the lung.

(A) Equivalent integrative visualization to Figure 6 for alternative examples of lineages located in the right lung of mouse MD7047. All microbiopsies from the trachea and the right lung containing the yellow clone (lineage) are shown as grey circles within the histological image. The phylogenetic tree depicted on the right-hand side is scaled according to the number of mutations per clone. The yellow ancestor and all subclones related to this clade are displayed. The small tree schematic surrounding the histological image is equivalent in structure, but not

scaled to the mutation burden of each clone. Each dot on the small tree represents a clone and branching point within the phylogeny.

(B) Equivalent to (A) but for all samples from the trachea and left lung of mouse MD7047 containing the black clone (lineage).

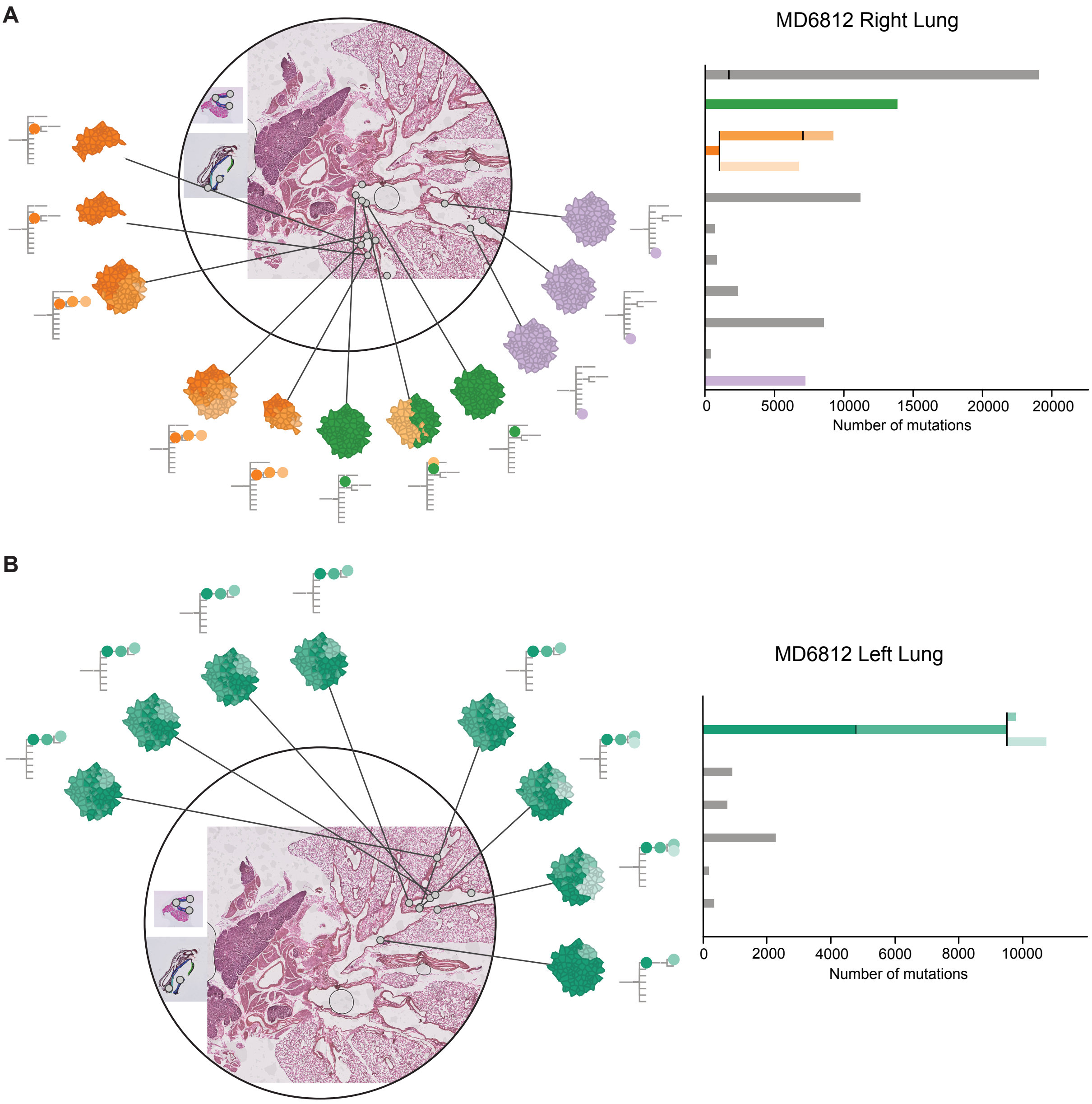

Fig. S11. NTCU-driven clonal expansions in the lung.
(A) Equivalent integrative visualization to Figure 6 for alternative examples of lineages located in the right lung of mouse MD6812. All microbiopsies from the trachea and the right lung containing the green, orange and lavender lineages are shown as grey circles within the histological image. The phylogenetic tree depicted on the right-hand side is scaled according to the number of mutations per clone. All subclones related to these clades and their ancestors are highlighted. The small tree schematic surrounding the histological image is equivalent in structure, but not scaled to the mutation burden of each clone. Each dot on the small tree represents a clone and branching point within the phylogeny.
(B) Equivalent to (A) but for all samples from the trachea and left lung of mouse MD6812 containing the green clone (lineage).

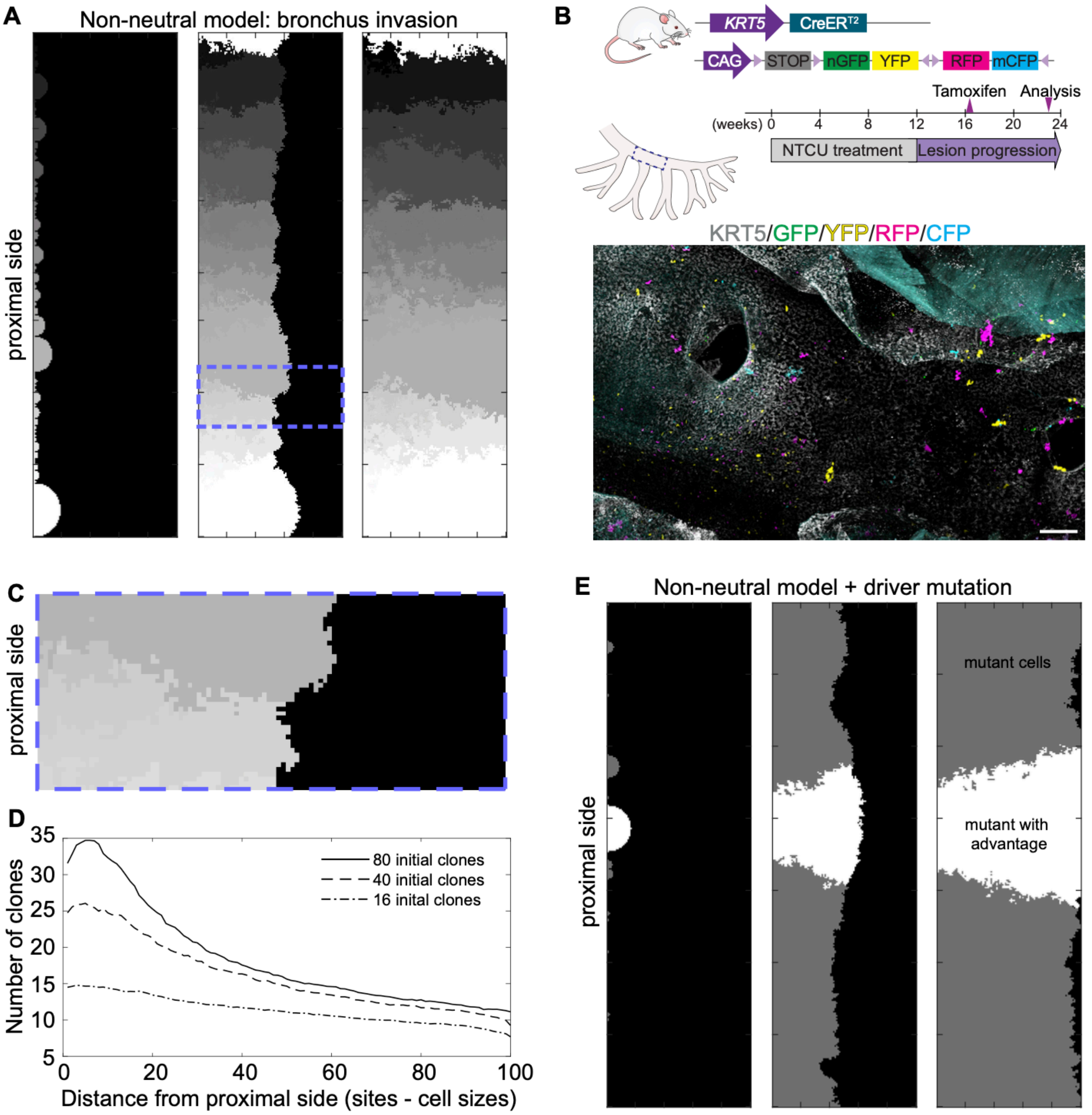

Fig. S12. **Basal cell colonization of the bronchial tree.**

(A) Representative images of a simulation of the non-neutral model in the bronchus (system size 100 × 350 cells) at three different times (increasing from left to right), here 80 individual clones were seeded on the proximal (left) side. Shades of gray correspond to distinct clones (for details see Supplementary Text).

(B) Clonal tracking of *Krt5*-expressing cells during NTCU-induced aberrant basal cell expansion in the bronchial tree. *Krt5-CreER*;*R26R-Confetti* mice were treated with NTCU for 12 weeks. Four weeks after NTCU treatment completion, mice received a daily dose of tamoxifen for 3 consecutive days to clonally label *Krt5*-expressing cells. 3D projection of lung whole-mount shows Confetti^{+} clones within the expanding KRT5^{+} domain (white staining) along the main bronchus, 23 weeks after NTCU commencement. Scale bar, 200 µm.

(C) Closeup of the region highlighted in (A), center panel showing the rough leading edge and fragmentation of clones due to competition.

(D) Decay of the number of distinct clones from proximal to distal regions of the airway obtained from numerical simulations of the non-neutral model, considering different numbers of seeded clones and measured at the time when clones span the whole airway (see, for example (A), right panel).

(E) Representative images of a simulation of the non-neutral model with driver mutations in the bronchus (system size 100 × 350 cells) at three different times (increasing from left to right), here 8 individual clones were seeded on the proximal (left) side, with the white clone having an advantage over its neighboring mutant clones. In (A), (C) and (E) the black background corresponds to normal cells.

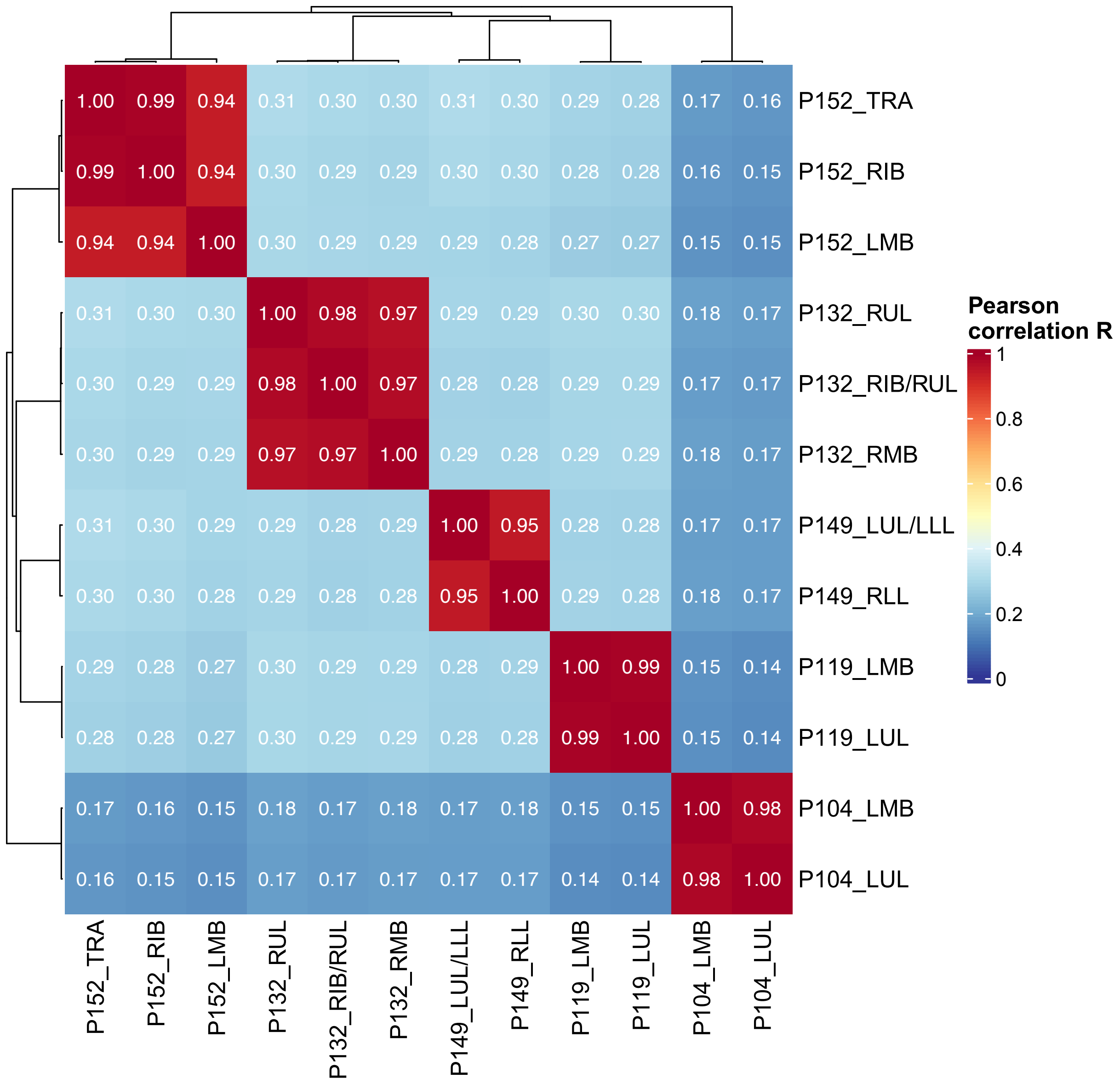

Fig. S13. Correlation heatmap of genotypes of human preinvasive lesions.
Heatmap illustrating sample-sample correlation analysis of sequenced human preinvasive lesions. Sample identity verification of WES data was performed by comparing genotypes at known SNP loci mapped to the hg19 reference genome. Pearson's correlation coefficient (R) was calculated for these SNPs to quantify genetic relatedness between samples. A threshold of $R \geq 0.90$ was used to identify samples from the same patient, accounting for the high genomic instability characteristic of preinvasive lesions.

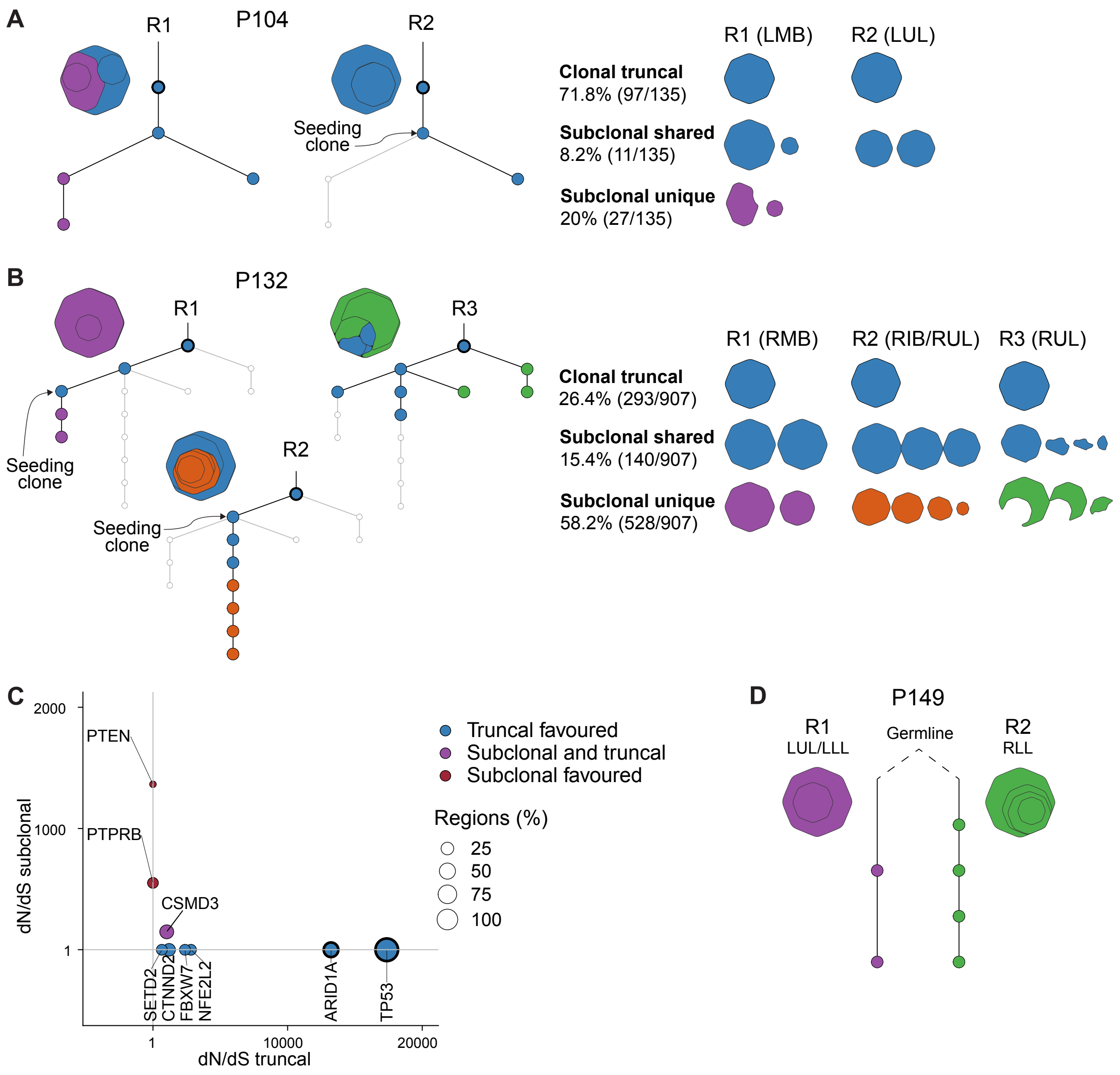

Fig. S14. Phylogenetic analysis of anatomically separate preinvasive lung lesions.
(A) Phylogenetic trees based on somatic mutations illustrating clonal relationships and evolutionary history between anatomically distinct lesions in a former-smoker with indolent lesions (P104).
(B) Phylogenetic reconstruction illustrating clonal relationships and evolutionary history between anatomically distinct lesions in a former-smoker with progressive lesions (P132). In (A) and (B) shared clusters across anatomical sites are colored in blue, while unique and site-specific clusters are colored in purple, orange, or green.
(C) Gene-level analysis of point mutation selection based on dN/dS ratios, comparing truncal and subclonal mutations in all patients with clonally-related lesions. Lung cancer driver genes were selected when dN/dS > 1; a dark black border indicates a global q-value < 0.1
(D) Phylogenetic tree based on somatic mutations present in a patient with progressive (R1) and an indolent (R2) lesions with no shared mutations between them.

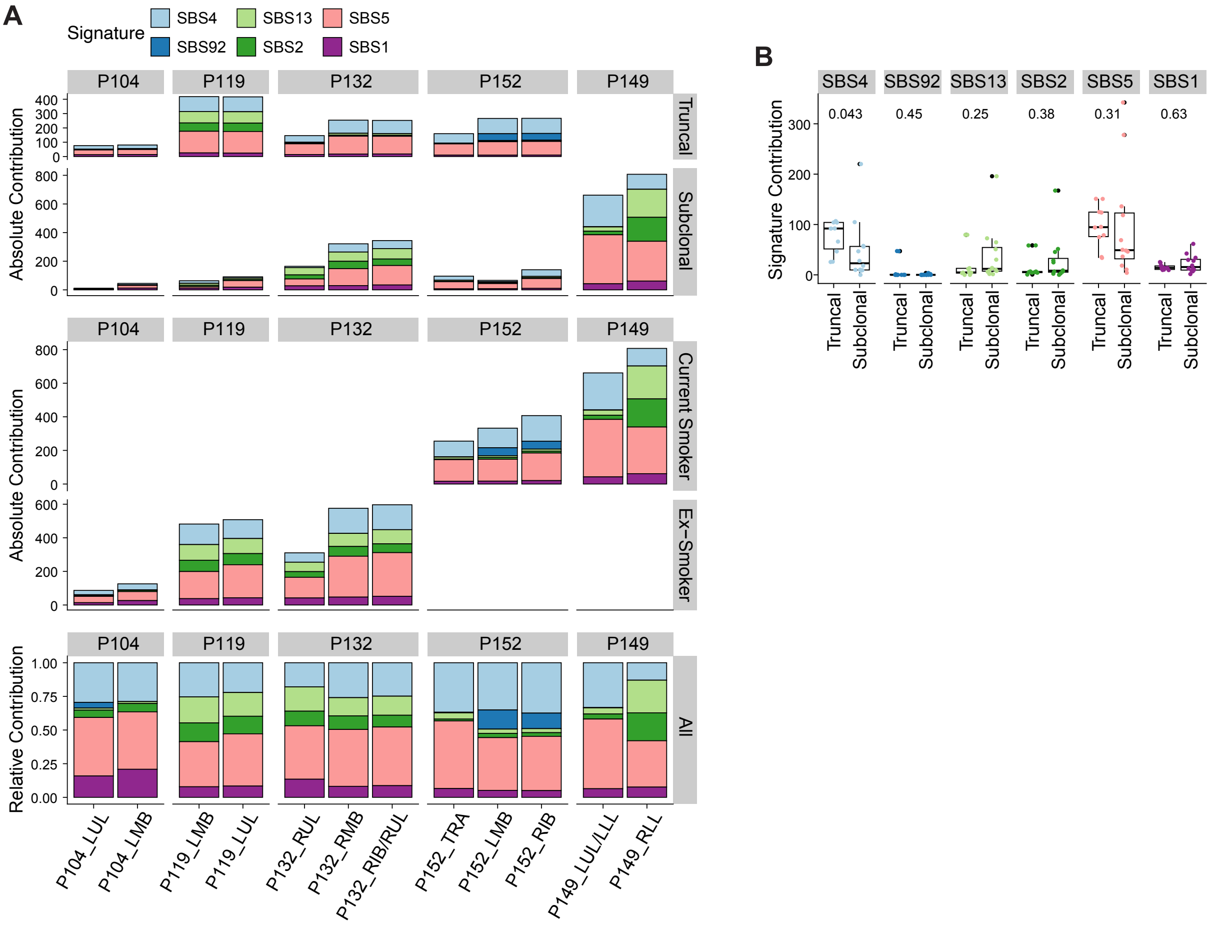

Fig. S15. Mutational signatures in human preinvasive airway lesions.
(A) Distribution of top six mutational single base substitutions (SBSs) signatures associated with lung carcinoma in situ (CIS) and LUSC across 5 patients with multi-site preinvasive high-grade lesions.
(B) Comparisons of absolute contribution of selected mutational signatures to truncal and subclonal mutations across all patients included in A.

Table S1.

List of antibodies.

Antibody	Source	Catalogue number
Mouse anti-FOXJ1	eBioscience	14-9965-82
Chicken anti-KRT5	BioLegend	905901
Rabbit anti-KRT5	BioLegend	905501
Rabbit anti-KRT13	Abcam	ab92551
Rabbit anti-KRT14	BioLegend	905301
Rabbit anti-Ki67	Thermo Scientific	RM-9106-S0
Rat anti-p63 (ΔN)	BioLegend	699501
Goat anti-SCGB1A1	Santa Cruz Biotechnology	sc-9772
Mouse anti-SCGB1A1	Santa Cruz Biotechnology	sc-365992
Rabbit anti-SCGB1A1	Merck Millipore	07-623
Mouse anti-acetylated-alpha Tubulin	Sigma Aldrich	T6793
Rabbit anti-alpha Tubulin (acetyl K40)	Abcam	ab179484
Mouse anti-Red Fluorescent Protein	Invitrogen	MA515257
Rabbit anti-Red Fluorescent Protein	Rockland	600-401-379
Donkey anti-chicken Alexa Fluor 488	Jackson ImmunoResearch	703-545-155
Donkey anti-chicken Alexa Fluor 647	Jackson ImmunoResearch	703-605-155
Donkey anti-goat Alexa Fluor 647	ThermoFisher	A21447
Donkey anti-goat Alexa Fluor Plus 647	ThermoFisher	A32849
Donkey anti-rabbit Alexa Fluor 488	ThermoFisher	A21206
Donkey anti-rabbit Alexa Fluor 555	ThermoFisher	A31572
Donkey anti-rabbit Alexa Fluor Plus 647	ThermoFisher	A32795
Donkey anti-rat DyLight 650	ThermoFisher	SA5-10029
Goat anti-mouse IgG1 Alexa Fluor 488	ThermoFisher	A21121
Goat anti-mouse IgG1 Alexa Fluor 555	ThermoFisher	A21127
Goat anti-mouse IgG2b Alexa Fluor 488	ThermoFisher	A21141
Horse anti-rabbit HRP ImmPRESS	Vector Laboratories	MP-7401

Data S1.
Murine trachea single-cell RNA sequencing statistics, including the number of cells and average number of genes per cell.

Data S2.
Cell type signatures from single-cell RNA-sequencing studies from the literature used for the annotation of murine cells.

Data S3.
Genes enriched in mouse tracheal epithelial cell clusters identified by scRNA-seq.

Data S4.
Human sample information. Description of human samples used for single-cell RNA and whole-exome sequencing studies.

Data S5.
Human reference gene lists. Cell type signatures from previous single-cell RNA-sequencing studies used for the annotation of human cell clusters. List of potential lung cancer driver genes curated from the literature.

Data S6.
Genes enriched in human tracheal epithelial cell clusters identified by scRNA-seq.

Data S7.
Summary of WGS data including information on all mouse microbiopsies.

Data S8.
Summary statistics of inferred clonal populations from NDP across both MD6812 and MD7047.

Data S9.
Interactive visualization of clones identified in MD7047 (A).

Data S10.
Interactive visualization of clones identified in MD7047 (B).

Data S11.
Interactive visualization of clones identified in MD6812 (A).

Data S12.
Interactive visualization of clones identified in MD6812 (B).

Data S13.
List of somatic mutations identified in human WES studies, with annotated known lung cancer driver genes.
