# Supplementary material for: Aberrant basal cell clonal dynamics shape early lung carcinogenesis
Source: Science. Author manuscript; Available in PMC 2025 Jun 23. (PMC7617789; doi:10.1126/science.ads9145)
Supplement: Supplementary References [file EMS206506-supplement-Supplementary_References.pdf]

69. M. Jamal-Hanjani *et al.*, Tracking the Evolution of Non-Small-Cell Lung Cancer. *The New England journal of medicine* **376**, 2109-2121 (2017).
70. F. A. Wolf, P. Angerer, F. J. Theis, SCANPY: large-scale single-cell gene expression data analysis. *Genome biology* **19**, 15 (2018).
71. M. D. Young, S. Behjati, SoupX removes ambient RNA contamination from droplet-based single-cell RNA sequencing data. *Gigascience* **9**, (2020).
72. C. S. McGinnis, L. M. Murrow, Z. J. Gartner, DoubletFinder: Doublet Detection in Single-Cell RNA Sequencing Data Using Artificial Nearest Neighbors. *Cell Syst* **8**, 329-337.e324 (2019).
73. H. Roux de Bézieux, K. Van den Berge, K. Street, S. Dudoit, Trajectory inference across multiple conditions with condiments. *Nat Commun* **15**, 833 (2024).
74. H. Li, R. Durbin, Fast and accurate short read alignment with Burrows-Wheeler transform. *Bioinformatics* **25**, 1754-1760 (2009).
75. T. H. H. Coorens *et al.*, Inherent mosaicism and extensive mutation of human placentas. *Nature* **592**, 80-85 (2021).
76. F. Blokzijl, R. Janssen, R. van Boxtel, E. Cuppen, MutationalPatterns: comprehensive genome-wide analysis of mutational processes. *Genome Med* **10**, 33 (2018).
77. L. B. Alexandrov *et al.*, The repertoire of mutational signatures in human cancer. *Nature* **578**, 94-101 (2020).
78. I. Martincorena *et al.*, Universal Patterns of Selection in Cancer and Somatic Tissues. *Cell* **171**, 1029-1041.e1021 (2017).
79. T. Zhang *et al.*, Genomic and evolutionary classification of lung cancer in never smokers. *Nat Genet* **53**, 1348-1359 (2021).
80. Cancer Genome Atlas Research Network, Comprehensive genomic characterization of squamous cell lung cancers. *Nature* **489**, 519-525 (2012).
81. Cancer Genome Atlas Research Network, Comprehensive molecular profiling of lung adenocarcinoma. *Nature* **511**, 543-550 (2014).
82. S. Chen, Y. Zhou, Y. Chen, J. Gu, fastp: an ultra-fast all-in-one FASTQ preprocessor. *Bioinformatics* **34**, i884-i890 (2018).
83. H. Li *et al.*, The Sequence Alignment/Map format and SAMtools. *Bioinformatics* **25**, 2078-2079 (2009).
84. A. Mayakonda, D. C. Lin, Y. Assenov, C. Plass, H. P. Koeffler, Maftools: efficient and comprehensive analysis of somatic variants in cancer. *Genome Res* **28**, 1747-1756 (2018).
85. M. Westphal *et al.*, SMaSH: Sample matching using SNPs in humans. *BMC Genomics* **20**, 1001 (2019).
86. J. D. Campbell *et al.*, Distinct patterns of somatic genome alterations in lung adenocarcinomas and squamous cell carcinomas. *Nat Genet* **48**, 607-616 (2016).
87. V. Sood, T. Antal, S. Redner, Voter models on heterogeneous networks. *Phys Rev E Stat Nonlin Soft Matter Phys* **77**, 041121 (2008).
88. R. Erban, S. J. Chapman, *Stochastic Modelling of Reaction–Diffusion Processes*. Cambridge Texts in Applied Mathematics (Cambridge University Press, Cambridge, 2020).

89. P. L. Krapivsky, Kinetics of monomer-monomer surface catalytic reactions. *Phys Rev A* **45**, 1067-1072 (1992).

Supplementary Materials

Materials and Methods

Supplementary Text

Figs. S1 to S15

Table S1

Data S1 to S13

References (*69-89)*
